# A multifunctional ultra-thin acoustic membrane with self-healing properties for adaptive low-frequency noise control

**DOI:** 10.1038/s41598-022-22441-4

**Published:** 2022-10-22

**Authors:** Marco Boccaccio, Konstantinos Myronidis, Michael Thielke, Michele Meo, Fulvio Pinto

**Affiliations:** Department of Mechanical Engineering, University of Bath, Bath, BA27AY UK

**Keywords:** Mechanical engineering, Gels and hydrogels, Acoustics

## Abstract

This paper proposes a novel multifunctional ultra-thin membrane based on a Polyborosiloxane-based gel with stimuli-responsive sound absorption and sound transmission loss (STL) and characterised by excellent self-healing properties. This adaptive behaviour is the result of a dynamically activated phase transition in the membrane’s polymeric network which is given by the interaction with the travelling sound pressure wave. The presence and the extent of such phase transition in the material was investigated via oscillatory rheological measurements showing the possibility to control the dynamic response by modifying the Boron content within the polymer. Acoustic analyses conducted at different stimuli responses showed high and dynamic absorption (95%) at the absorption coefficient peaks and an adaptive shift to lower frequencies while sound amplitudes were increased. An average STL up to 27 dB in the frequency range between 500 to 1000 Hz was observed and an increased STL above 2 dB was measured as the excitation amplitude was increased. Results demonstrated that the new membrane can be used to develop deep subwavelength absorbers with unique properties (1/54 wavelength in absorption and 1/618 in STL) able to tune their performance in response to an external stimulus while autonomously regaining their properties in case of damage thanks to their self-healing ability.

## Introduction

Effective low frequency sound absorption and transmission loss have posed an issue for decades and consequently has attracted the attention of researchers for physics and engineering applications. The most prominent area of investigation has been on composite materials for sound absorption applications^1,2^, with panels composed of synthetic fibres, mineral- or glass-fibre^3–5^, which are currently commercially available. However, the employment of these materials has grown concerns in health and environmental safety, due to the potential health hazards associated with the exposure of these fibres to humans. As a result of this limitation, the focus of researchers has turned on the employment of agricultural natural fibres, which are renewable and pose a low-health risk, as a replacement of synthetic fibres in composite materials and wood-based components for acoustic purposes^6–8^. Furthermore, researchers have also focused their effort on the development of alternative sound absorbing structures from recycled materials, such as foam rubbers or textiles to achieve good acoustic properties in a more environmentally friendly manner^9–11^. Although products made from both renewable and recycled materials are characterised by relatively good acoustic properties, the energy consumption during manufacturing of these structures has limited their applications^12^.

Polymeric materials have been widely applied for noise reduction and sound absorption purposes, due to their viscoelastic properties and commercial availability^13–15^, with results showing good sound absorption performances, which descend from the link between the visco-thermal resonating mechanisms of the polymeric structures and damping effects^14^. Several polymers have been tested as sound absorption materials, including polyacrylic ester, polystyrene, multifunctional polyethylene and polyurethane, however, even if they all exhibit high sound absorption coefficients^16–19^, these synthetic and plastic absorbers generally lack low frequency absorption properties, since sound attenuation can be described as a quadratic function of the frequency^20^. In this context, vibrating acoustic metamaterials and coherent absorbers have been proposed to achieve acceptable absorption properties in the low frequency range^21–23^, however they tend to only restrict a narrow frequency bandwidth around their first resonance mode. To overcome this limitation, acoustic membrane-type metamaterial structures have been proposed to achieve nearly full absorption at the resonance frequency, due to the flapping motion of non-symmetrical rigid platelets introduced to the membranes^24,25^. However, since the lower frequency is imposed by the material thickness, they become unfeasible for noise reduction in construction and building applications, where space, weight and thickness limitations are imposed. In addition, membrane-type sound absorbers are vulnerable to mechanical loads or high sound pressure which can damage the substrate, if unprotected and directly exposed, resulting in poor acoustic performance due to a variation of the vibrational response of the membrane^26,27^.

High-efficiency acoustic metamaterials have emerged to provide an alternative way to develop novel absorbers, due to the resonance modes of these structures at lower frequencies^28,29^. Perforated panel absorbers have been developed to provide a robust fibre-free alternative to conventional sound absorption materials relying on the perforation of a plate positioned in front of a rigid back wall with an air cavity between them, to broaden the absorbed frequency bandwidth^30–36^. Though these perforated sound absorbers may overcome some of the inherent disadvantages of porous materials-based sound absorbers^36^, their absorption performance is static and limited at a certain frequency range, and more importantly, since their performance is highly dependent on the geometrical properties, low-frequency absorption can be achieved only with large and unfeasible thicknesses^37^. Considering the drawbacks of the current solutions, a demand for alternative low-cost and effective materials, that pose no threat to health are required.

This work focuses on the development of a novel ultrathin Polyborodimethylsiloxane (PBDMS) based pseudo non-Newtonian acoustic membrane gel (nNAMeG), for achieving dynamic sound absorption and transmission loss mechanisms, that surpasses all the aforementioned limitations, allowing high-efficiency autonomous control of its acoustic response at low frequencies. In addition to its excellent acoustic performance, the self-healing ability of the material enables full restoration of its properties resulting in a complete multifunctional solution for industrial applications. The self-healing abilities of the PBDMS material are on account of the dynamic and reversible non-covalent B-O bond and the molecular mobility of the polymer, with spontaneous healing occurring without any external catalysts and have been documented in literature^38^. In particular, Wu et al.^39^ have formulated a “solid–liquid” elastomer by interpenetrating PDMS with PBS and cuts were introduced to their material which healed completely within 24 h. Post the incisions, they evaluated the tensile properties of the healed elastomer with results suggesting approximately full restorability of the elastomer’s mechanical properties in this time frame. In a study by Wang et al.^40^ PBDMS was employed into a polyurethane sponge in an attempt to create a flexible body armour; in order to demonstrate the self-healing abilities of the material the authors introduced cuts to the sponge, which they then simply pushed together with the individual parts adhering to each other. Since their experiments focused on safeguarding, they measured the rebound energy prior and post the cuts of the sponges with values being at very close proximity to the pristine samples. Finally, D’Elia et al.^41^ performed a series of mechanical experiments to a PBS-based material, demonstrating that the material has the ability to successfully self-heal multiple times at room temperatures autonomously.

Firstly, a chemical characterisation was conducted via means of Fourier transform infrared spectroscopy, followed by rheological measurements to provide a mechanical characterisation of the gels and evaluate the presence and the extent of the pseudo non-Newtonian mechanism. Thereafter, absorption and sound transmission loss analyses were conducted on nNAMeGs, to characterise the acoustic properties of the material and demonstrate their unique and unprecedented performance as human-and-environmentally safe, adaptive and ultra-thin acoustic material, with the target of showing above 95% absorption with thicknesses up to 1/54 wavelength and sound losses above 20 dB with extremely small thicknesses up to 1/618 wavelength. Finally, the self-healing properties of the proposed material were demonstrated via microscopic and acoustic analyses.

## Results

### Synthesis of polyborodimethylsiloxane with boric acid

The main concept of the nNAMeG is to exploit its dynamic change in stiffness, owed to the phased transition from viscous to rubbery state, to modulate the sound absorption characteristics and control this change via tailoring the structure of the membrane according to the needs of the specific application. Materials exhibiting a divergence from the traditional Newtonian behaviour with a linear increase of viscosity in response to an applied load, have attracted great attention and in particular, materials which display a rapid, non-linear increase of viscosity for the same magnitude of stress, have been employed in a wide range of applications^42–44^. Shear Thickening Fluids (STFs) were developed, based on colloidal solutions that are characterised by large changes in viscosity which result from the formation of “hydroclusters” due to the particle–particle interactions response to an external load^45^. The liquid nature of the STFs has however restricted their potential applications, thus materials with a more stable nature have emerged, such as Shear Stiffening Gels (SSG). A more detailed description of the stiffening mechanism of SSGs can be found in Supplementary Material. In this work, the unique shear dependent behaviour of nNAMeGs was exploited to develop a novel acoustic material to drastically reduce sound in the low frequency range with capability to dynamically respond to an external stimulus. The synthesis of PBDMS-based SSGs has been previously reported by our group^46,47^. In detail, the first step of the synthesis of the nNAMeG s was the conversion of boric acid $({\mathrm{H}}_{3}{\mathrm{BO}}_{3})$, firstly at metaboric acid ${(\mathrm{H}}_{3}{\mathrm{B}}_{3}{\mathrm{O}}_{6})$ at approximately 100 ℃, as seen below.

$$3{\mathrm{H}}_{3}{\mathrm{BO}}_{3}\stackrel{100\mathrm{^\circ{\rm C} }}{\to } {\mathrm{H}}_{3}{\mathrm{B}}_{3}{\mathrm{O}}_{6}+ {3\mathrm{H}}_{2}\mathrm{O}$$

In the second step of the reaction, the formed ${\mathrm{H}}_{3}{\mathrm{B}}_{3}{\mathrm{O}}_{6}$ dehydrates to pyroboric acid $({\mathrm{H}}_{2}{\mathrm{B}}_{4}{\mathrm{O}}_{7})$, at approximately 140 ℃, as seen below.

$$4{\mathrm{H}}_{3}{\mathrm{B}}_{3}{\mathrm{O}}_{6}\stackrel{140\mathrm{^\circ{\rm C} }}{\to } 3{\mathrm{H}}_{2}{\mathrm{B}}_{4}{\mathrm{O}}_{7}+ 3{\mathrm{H}}_{2}\mathrm{O}$$

**Figure 1:**
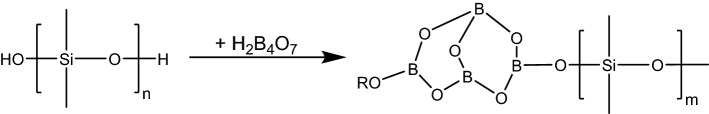
${\mathrm{H}}_{2}{\mathrm{B}}_{4}{\mathrm{O}}_{7}$ insertion into PDMS polymeric backbone.

In the final step of the synthesis, ${\mathrm{H}}_{2}{\mathrm{B}}_{4}{\mathrm{O}}_{7}$, is inserted into the polymer backbone (non-stoichiometric conversion), resulting in the PBDMS as shown in Fig. 1.

**Figure 2:**
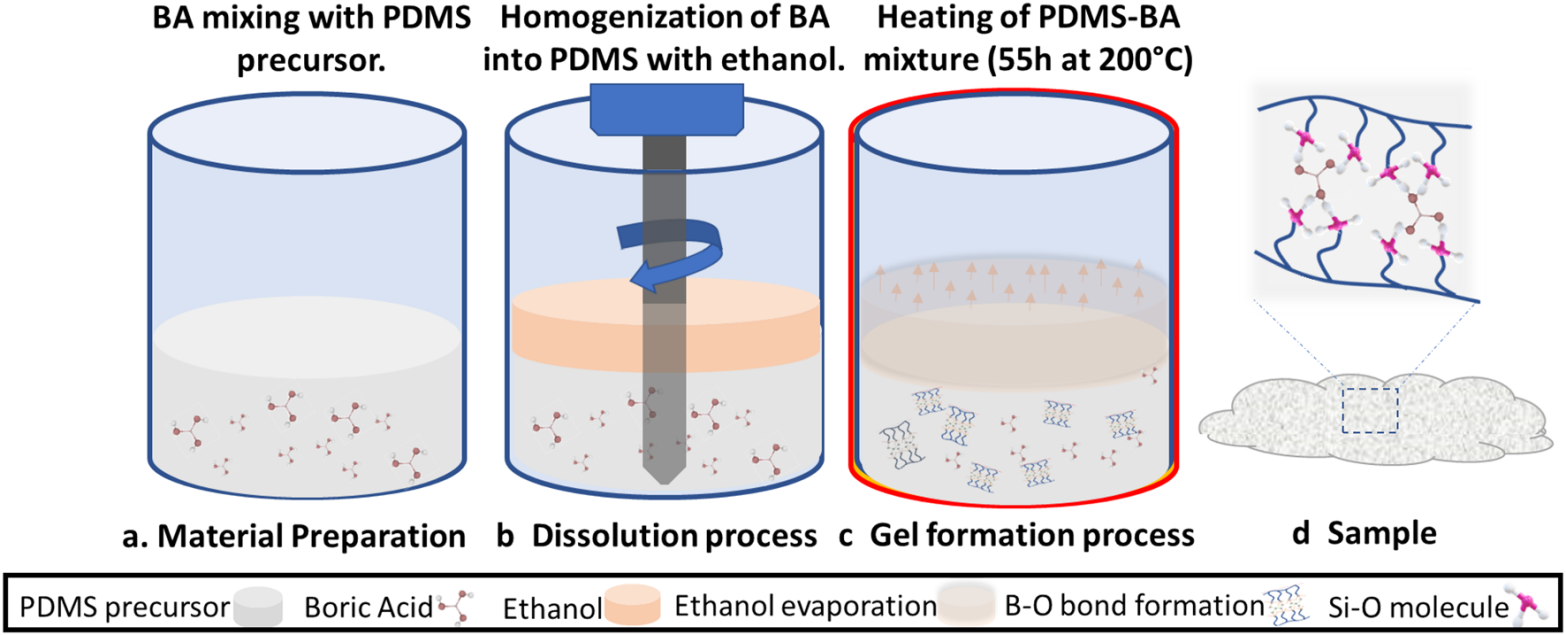
Schematic representation of the manufacturing process of nNAMeGs.

**Figure 3:**
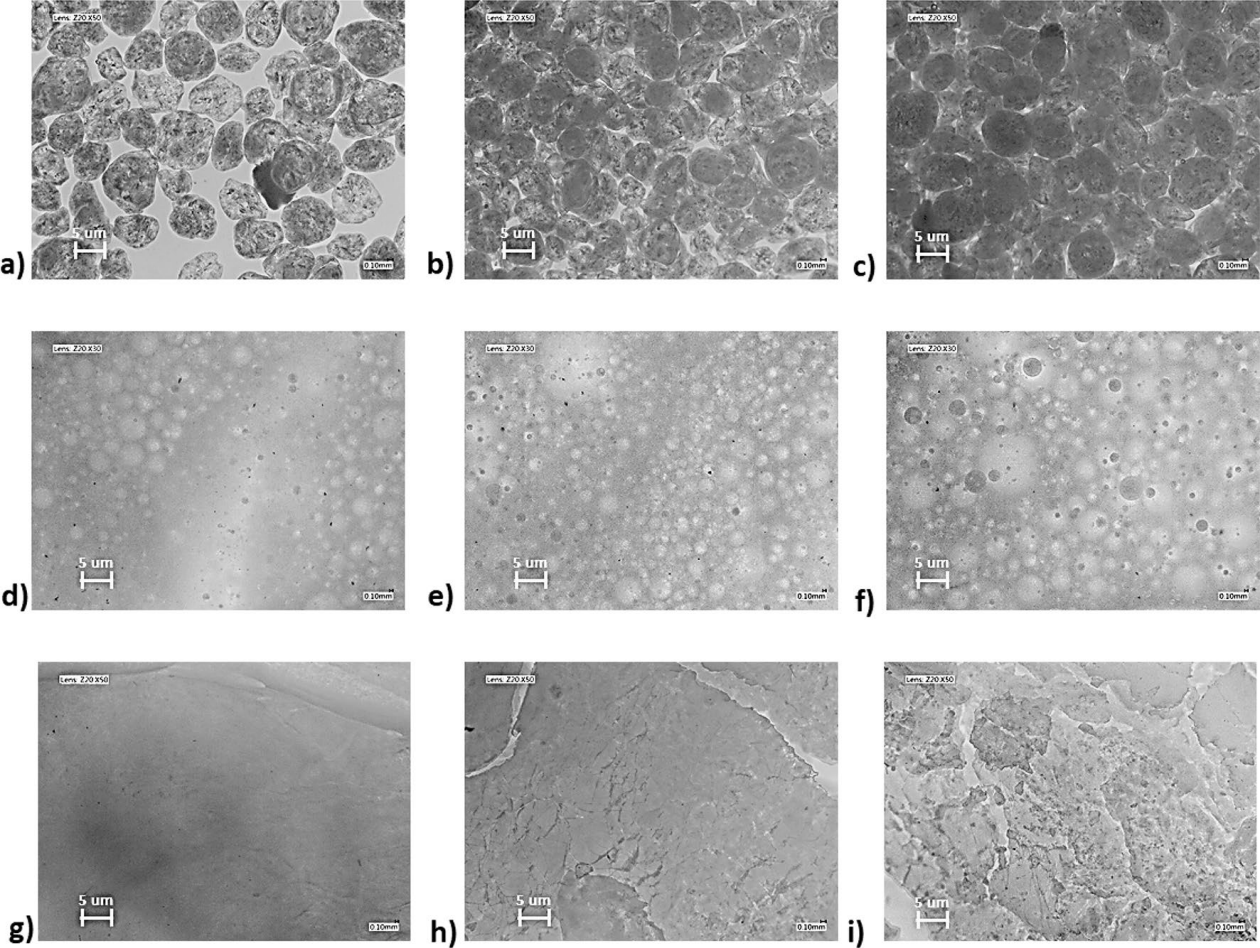
OLM imaging of the nNAMeGs manufacturing process: (**a–c**) BA mixing with PDMS precursor; (**d–f**) Homogenization of BA into PDMS; (**g–i**) final NNAMeG samples. Different stoichiometric Boron ratios: (**a,d,g**) ${X}_{BA}^{st}$ = 0.2 (NNAMeG02); (**b,e,h**) ${X}_{BA}^{st}$ = 0.5 (NNAMeG05); (**c,f,i**) ${X}_{BA}^{st}$ = 0.8 (NNAMeG02). Magnification: (**d–f**) × 30, (**a–c,g–i**) × 50.

Various amounts of ${\mathrm{H}}_{3}{\mathrm{BO}}_{3}$ were used to synthesise nNAMeGs with stoichiometric ratios of Boron to Silicone of 0.2 (5.0 g), 0.5 (12.5 g), and 0.8 (20.0 g) with a schematic of the synthesis shown in Fig. 2. Figure 3 provided an Optical Light Microscopy (OLM) step-by-step investigation of the manufacturing process. As shown in OLM imaging in Fig. 3g–i, these result in a cloudy colourless viscoelastic material which was employed in the measurements presented in this work without any further treatment. The greater amounts of ${\mathrm{H}}_{2}{\mathrm{B}}_{4}{\mathrm{O}}_{7}$, would lead in an abundance of hydroxyl groups in the siloxane chains, thus low, intermediate and high Boron/Silicone ratios were explored to investigate the effect of the amount of supramolecular bonds to the acoustic properties of the material, in addition to the various excitation levels. Pyroboric Acid was dispersed in 30.0 g of Polydimethylsiloxane (PDMS) with a viscosity of 200 cSt. 10 mL of ethanol were added to the dispersion to improve the homogenisation of the solution, which was then heated to 200 °C for 55 h.

### Chemical characterisation of PDMS-PBDMS conversion via Fourier transform-infrared spectroscopy (FT-IR)

**Figure 4:**
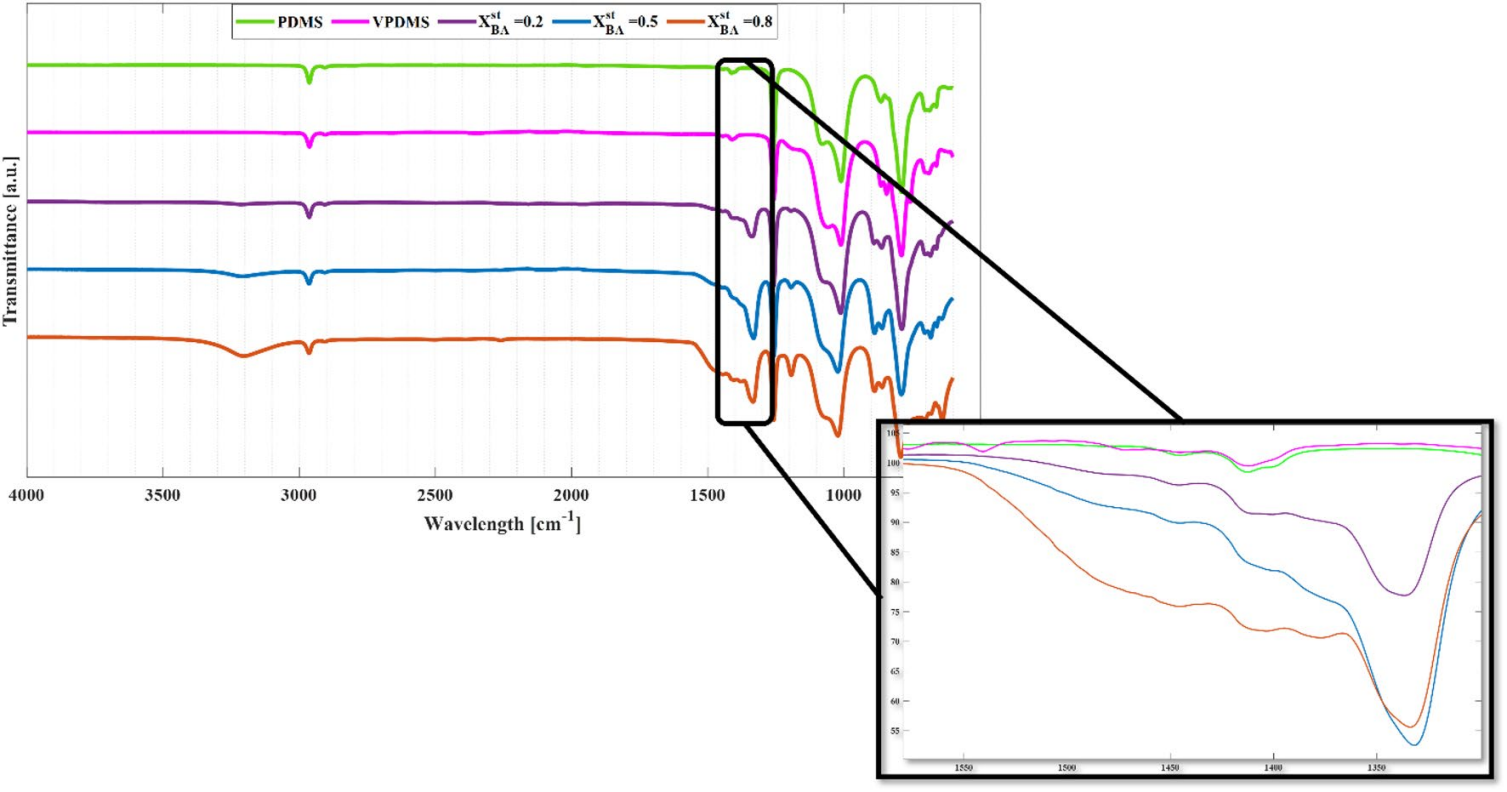
FT-IR spectra of PDMS precursor (green line), vinyl terminated PDMS (purple line) and samples synthesised with various stoichiometric ratios of boron to silicone. Insert in figure focusing around area of interest at approximately 1300 cm^−1^.

The conversion of PDMS to PBDMS can be established via FT-IR spectroscopy. The spectra in Fig. 4 derive from the PDMS precursor and the resulting PBDMS of various boron ratios. In addition, the spectra of a traditional vinyl terminated PDMS (Sylgard 184) have also been included, as an additional comparative material. In good agreement with literature^48–50^, both PDMS (green line) and VPDMS (purple line) exhibited significant peaks approximately at 2900 cm^−1^ (C-H, s) stretch, 1400 cm^−1^ (C-H, w) deform, 1250 cm^−1^ (Si-CH_3_, s) stretch, 1100 cm^−1^ (Si–O-Si, s) stretch, 1000 cm^−1^ (Si–O-Si, s) vibrations and 700 cm^−1^ (Si-CH_3_, s) stretch. In comparison, the PBDMS samples (red, blue and black lines) exhibited two additional significant peaks, at 3200 cm^−1^ (O–H, broad peak) stretch and 1380 cm^−1^ (B-O-B, s) stretch^51,52^, with the intensity of these peaks directly related to the stoichiometric ratio of boron employed. The hydroxy stretching at 3200 cm^−1^ compared to the signal of 2900 cm^−1^ can be ascribed to methyl groups. The boron oxygen stretch at 1380 cm^−1^ can be an important indicator for the reaction of the boric acid with the PDMS^38^, however the appearance of this peak can be from traces of boric acid or PBDMS^51^. Considering the low intensity of the O–H stretch signal at 3200 cm^−1^ in combination with the B-O-B stretch signals at 1380 cm^−1^, it is possible to conclude that there is a conversion of the boric acid into the PBDMS. As the intensity of the latter stretch signal (see insert Fig. 4) is directly related to the amount of boron employed in the synthesis of the PBDMS, it can be assumed that greater amounts of boron would lead to an abundance of dynamic and reversible bonds of boron-oxygen, which in return will promote the self-healing capabilities of the PBDMS.

### Rheological characterisation of the nNAMeG materials

The nNAMeGs presented in this work are viscoelastic materials, exhibiting elastic and viscous behaviours over different time scales; their unique behaviour is given by the fact that after a critical strain is applied, they respond macroscopically by increasing their stiffness behaving like a solid and absorbing more energy in the process. In order to quantify this effect and analyse its dependence with the increasing amount of B-O bonds, oscillatory rheological measurements can be employed to investigate the transition from viscous dominating to elastic dominating properties of nNAMeG, measuring the change in the storage $\left( {G^{^{\prime}} } \right)$ and loss moduli $\left( {G^{^{\prime\prime}} } \right)$ , representing their elastic and viscous behaviour respectively.

**Figure 5:**
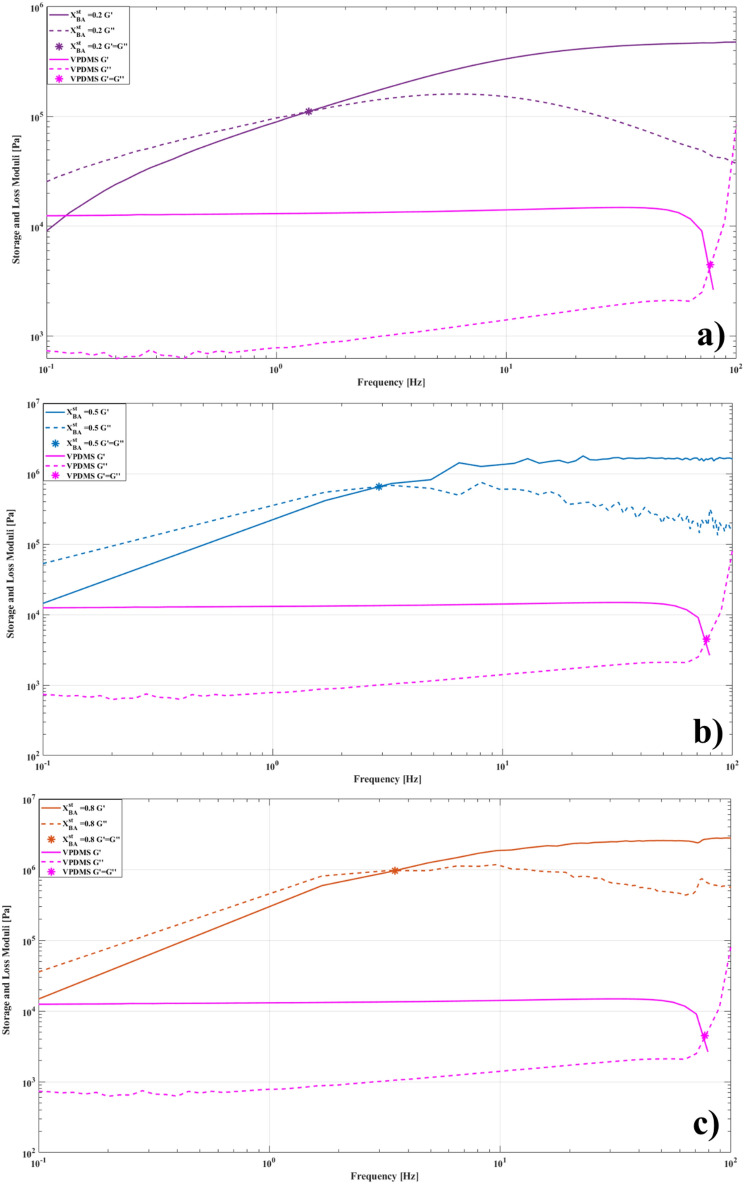
Rheological Measurements of nNAMeGs samples and comparison with VPDMS; (**a**) ${\mathrm{X}}_{\mathrm{BA}}^{\mathrm{st}}=0.2$; (**b**) ${\mathrm{X}}_{\mathrm{BA}}^{\mathrm{st}}=0.5$; (**c**) ${\mathrm{X}}_{\mathrm{BA}}^{\mathrm{st}}=0.8.$

In case of SSGs, it is evident that a change in their internal structure happens when the value of the G’ surpasses that of G’’, indicating an elastic-dominated behaviour, therefore it is possible to identify the critical dynamic point associated with the phase transition as the frequency for which G’ = G’’. Additional considerations on rheological measurements and the relationship of the dynamic moduli to the phase transitions the SSGs exhibit can be found in the Supplementary Information. The rheological measurements of the nNAMeGs prepared with three different ratios of Boron to Silicone ${\mathrm{X}}_{\mathrm{BA}}^{\mathrm{st}}$ are represented in Fig. 5, where the continuous lines illustrate the storage moduli of the samples and the dashed lines their loss moduli, respectively. The nNAMeG synthesised with ${\mathrm{X}}_{\mathrm{BA}}^{\mathrm{st}}$=0.2 is depicted in red, while the nNAMeG with ${\mathrm{X}}_{\mathrm{BA}}^{\mathrm{st}}$=0.5 is in blue, and finally the one prepared with ${\mathrm{X}}_{\mathrm{BA}}^{\mathrm{st}}$=0.8 is represented by the black line. The coloured markers indicate the moduli intersection per ratio, which corresponds to the liquid/solid transition. The performance of nNAMeGs was compared with traditional PDMS, in order to demonstrate how the dynamic behaviour of the NNAMeG membrane originates from the introduction of Boron within the polymeric chains of the PDMS. Table 1 contains tabulated data obtained from rheological measurements of samples.

**Table 1:** Rheological Measurements of NNAMeGs.

Material	Min. Gʹ [Pa]	Max. Gʹ [Pa]	∆ Gʹ [Pa]
VPDMS	2643	12,472	− 9829
nNAMEG $$X_{{}}^{{}}$$$${\text{X}}_{{{\text{BA}}}}^{{{\text{st}}}} \, = \,0.2$$	9064	474,952	466,773
nNAMEG ${\text{X}}_{{{\text{BA}}}}^{{{\text{st}}}} \, = \,0.5$	14,384	1,690,910	1,599,550
nNAMEG ${\text{X}}_{{{\text{BA}}}}^{{{\text{st}}}} \,{ = }\,{0}{\text{.8}}$	15,679	2,309,620	2,217,260

Analysing the results of Fig. 5, it is evident that while the traditional PDMS (purple line) displayed the typical Newtonian response of an elastomer, the nNAMeG samples displayed a non-linear behaviour and more specifically, a shift from viscous dominating properties to elastic dominating properties as the frequency was increased, indicating the presence of the shear stiffening effect^53^. This shift is evident in the intersection of the storage and loss moduli of each sample, at which, the samples’ elastic properties prevail their viscous ones^54^. The minimum values of storage moduli were also the initial values, designating soft gels at the frequency when the oscillatory tests were initiated. The maximum values of storage moduli were approximately 0.5, 1.7 and 2.3 MPa for the NNAMeGs prepared with a ratio of Boron to Silicone of 0.2, 0.5 and 0.8 respectively, displaying an increase of approximately 112% between 0.2 and 0.5 and a further increase of 31% between the 0.5 and 0.8 samples. The same pattern was observed with overall change of the storage moduli of the samples, where an increase of approximately 110 and 32% was detected, between corresponding samples. Since the PDMS precursor and synthesis process was kept constant for all nNAMeGs, the variation of moduli can only be attributed to the different stoichiometric ratios employed in the process. The values recorded for G’’ increased gradually approximately up to the intersection point of the moduli after which they followed a regressive trend. Increasing the amount of Boron leads to an abundance of dynamic crosslinks in the polymeric network of the PDMS precursor, with this increase resulting in a more profound stiffening effect. This is consequently translated into a higher performance of the nNAMeG, as the supramolecular network is able to absorb energy more efficiently due to the more pronounced phase transition of the material.

### Absorption measurements

**Figure 6:**
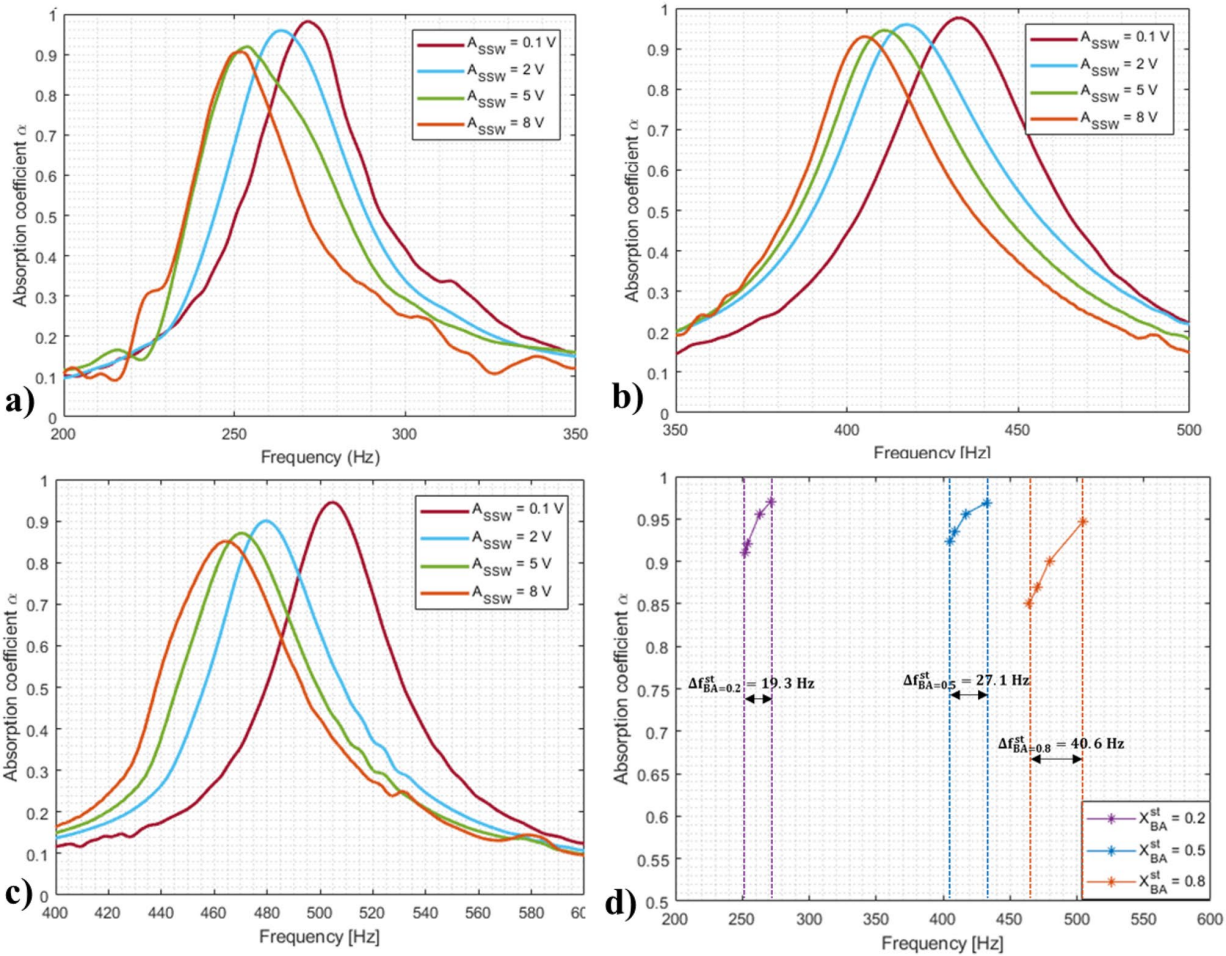
Absorption coefficient measurements, different voltage levels; (**a**) ${X}_{BA}^{st}=$ 0.2; (**b**) ${X}_{BA}^{st}=$ 0.5; (**c**) ${X}_{BA}^{st}=$ 0.8; (**d**) Comparison of sound absorption coefficient measured with different nNAMeGs.

For the sound absorption measurements, nNAMeG samples with a thickness of 1.1 mm were placed inside a 50.8 mm in diameter acrylic ring with a 25 mm air gap between the investigated material and the rigid wall on the tube. A 0.18 mm thickness substrate was employed to provide a consistent constrain to the gel, without affecting the performance of the test samples. For the acoustic tests, four different voltage levels of the sweep sinusoidal wave A_SSW_ were used, 0.1 mV, 2 V, 5 V and 8 V, in order to characterise the acoustic behaviour of the proposed gels when different sound pressure loads were applied. The results presented are average values of three measurements under the same conditions. The sound absorption α-coefficients were evaluated for each NNAMeG membrane and four different voltage levels. Figure 6 represents the absorption coefficients for the different excitation signals, showing absorption peaks that occur at the low-frequency range, between 200 and 600 Hz. Additionally, it is possible to see a clear shift in the frequency peaks at lower values as the voltage level is increased, as indicated by the numerical results in Table 2. In detail, Fig. 6a shows average absorption peaks around 260.2 Hz for 0.2, while the 0.5 in Fig. 6b presents an α-coefficient average peak at 416.56 Hz Finally, in Fig. 6c, the measured absorption coefficient for 0.8 shows the absorption maximum to average at 479.75 Hz.

**Table 2:** Average absorption frequency peaks evaluated for nNAMeG samples at different voltage levels (σ_α peak_ <  ± 0.02).

Boron ratio ${[\mathrm{X}}_{\mathrm{BA}}^{\mathrm{st}}]$	Peak for A_ssw_ = 0.1 V [Hz]	Peak for A_ssw_ = 2 V [Hz]	Peak for A_ssw_ = 5 V [Hz]	Peak for A_ssw_ = 8 V [Hz]
0.2	271.4	263.5	253.7	252.1
0.5	432.3	417.3	411.1	405.2
0.8	504.7	479.3	470.8	464.1

The results clearly show a dynamic and controllable shift of the absorption peaks toward lower frequencies as the voltage level increases. This is attributable to the evident stiffening effect of the pseudo non-Newtonian materials induced as the external stimulus (i.e., sound pressure wave) increases. The energy absorbed for the dynamic formation/crosslinking of the supramolecular B-O network and the decay of the thermal energy dissipation dynamically change the acoustic properties of the material, which are highly-affected by the dynamic non-acoustic transport parameters of the non-Newtonian material (i.e. tortuosity, characteristic length, and permeability), which depends on the current morphology of the polymeric network, and the thermal-dependent parameters (i.e. dynamic viscosity, thermal conductivity and specific heat capacity)^20,35,55–57^. Detailed description on the B-O network cross-linking mechanisms and thermal dissipation effect on the acoustic properties can be found in Supplementary Information.

Figure 6d shows the measured frequency peaks shift for each inspected sample for each excitation voltage levels. Results showed that the highest frequency shift was measured for ${\mathrm{X}}_{\mathrm{BA}}^{\mathrm{st}} =$ 0.8 (i.e. highest Boron ratio), which are also in good agreement with the rheological measurement, exhibiting the greatest shear stiffening effect. Taking into consideration that the PDMS precursor used for the formulation of the nNAMeGs was the same, it is evident that these demonstrate an adaptive behaviour which is driven by the excitation signal and that can be effectively tuned by the Boron/Silicone ratio. The former can be appreciated in the shift of absorption frequency under different voltage excitation levels, while the latter is evident in Fig. 6d, where a direct comparison between the samples is illustrated. Increasing the amount of Boron-Oxygen supramolecular cross bonds leads to a more efficient stiffening effect, as demonstrated by the increase in the slope of the rheological measurements, which translates to a more pronounced shift in frequencies. The magnitude of the absorption coefficients approaches unity for the vast majority of the results, denoting that most of the incident energy is absorbed by the material. The increase of this incident energy changes the frequency range where the sound absorption is achieved, further corroborating the dynamic nature of the nNAMeGs and their exclusive tuneable characteristics.

### Sound transmission loss measurements

**Figure 7:**
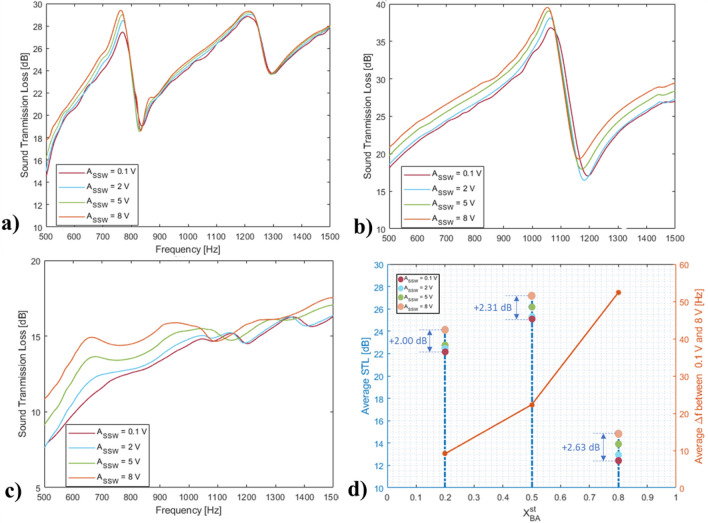
Sound Transmission Loss measurements, different voltage levels; (**a**) ${X}_{BA}^{st}=$ 0.2; (**b**) ${X}_{BA}^{st}=$ 0.5; (**c**) ${X}_{BA}^{st}=$ 0.8; (**d**) Average frequency shift in STL between ASSW = 0.1 V ASSW = 8 V.

Sound Transmission Loss (STL) were also investigated for the three nNAMeGs with four different sweep signals. Figure 7a depicts the STL measured for ${\mathrm{X}}_{\mathrm{BA}}^{\mathrm{st}}$ = 0.2, which shows an amount of sound being isolated by the novel material higher than 18 dB (i.e. 87% loss) for the majority of the measured low frequency range (500–1000 Hz), and approach 20 dB (90% loss) above 700 Hz. Moreover, an alternating trend of the curves was recorded, especially at low voltage levels, as well as a distinct STL dip around 750 Hz. These values represent the corresponding vibrational modes of the structure being inspected, and other minor vibrational effects due to the gel-like appearance of nNAMeGs. Similarly, STL evaluated from the tests conducted for ${\mathrm{X}}_{\mathrm{BA}}^{\mathrm{st}}$ = 0.5 are shown in Fig. 7b, where the measured STL is above 20 dB (84.1% loss) for the entire frequency range with an increasing trend, above 35 dB from 900 Hz. Figure 7c shows STL measured for ${\mathrm{X}}_{\mathrm{BA}}^{\mathrm{st}}$ = 0.8, where the loss exceeds 10 dB (68.9%) from 520 Hz, and linearly approach 15 dB at 1000 Hz. Additionally, the curves approach the 10 dB threshold only at 600 Hz. As revealed in the acoustic measurements, the dynamic effect of all nNAMeG membranes is evident on the shift of the curves across the low frequency range. Additionally, Fig. 7d shows the average frequency shift recorded in the STL measurements, where results from 0.8 show the highest frequency shift towards higher frequency, in a direct correlation with the results obtained from the rheological and absorption measurements. Transmission test results demonstrated an excellent attenuating behaviour of the novel material, with up to 90% of the propagating signal amplitude attenuated along a very large frequency bandwidth (i.e. from 500 Hz to above).

### Self-Healing properties of nNAMeG

**Figure 8:**
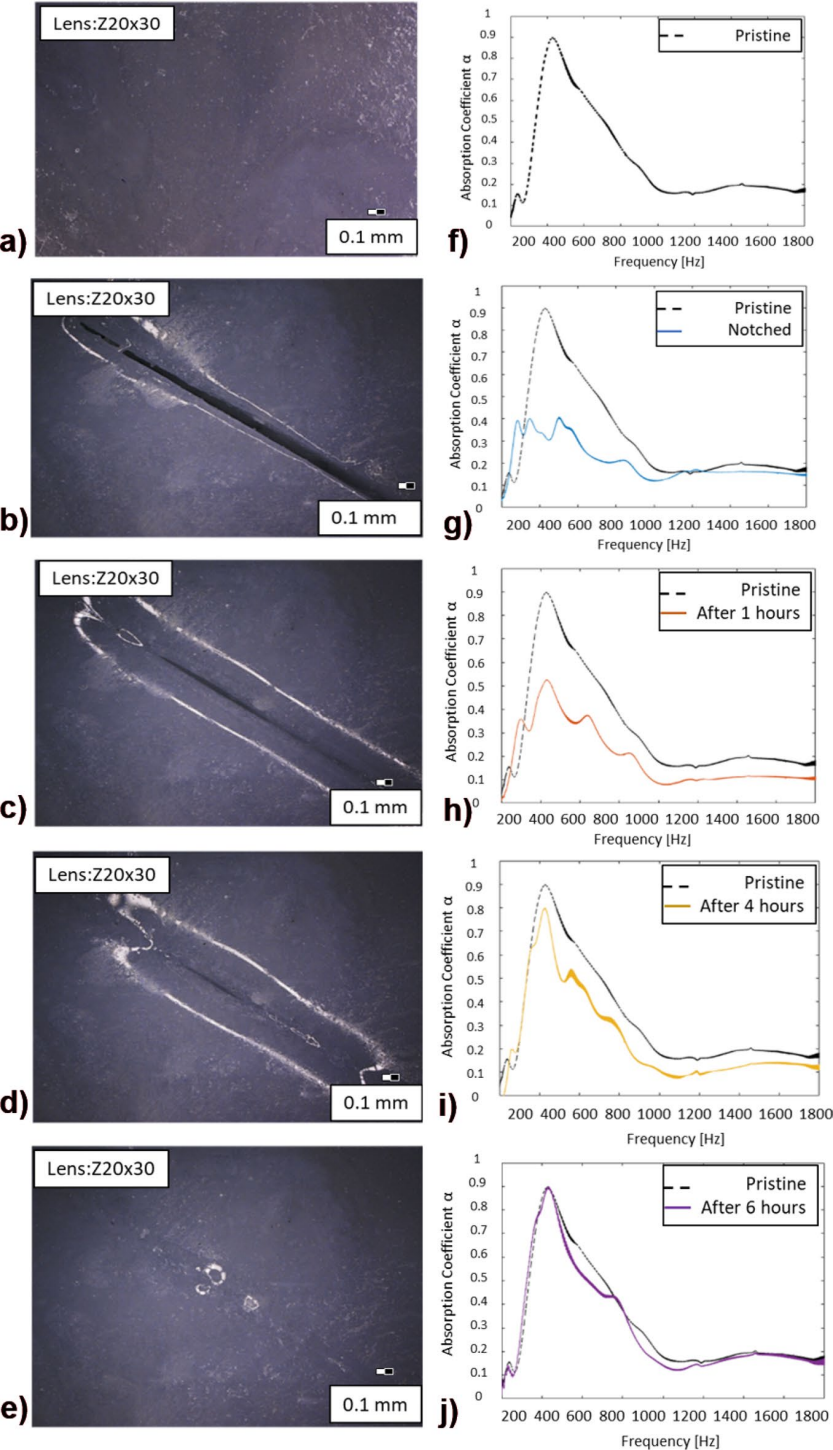
Time-lapse on the incised nNAMeG; (**a–e**) Optical light microscope imaging; (**f–i**) absorption coefficient measurements on the engraved nNAMeG in comparison to pristine sample; (**a,f**) before incision; (**b,g**) after initial incision; (**c,h**) after 1 h; (**d,i**) after 4 h; (**e,j**) after 6 h.

Intrinsic self-healing materials, namely those which do not require a separate healing agent to achieve this ability are sparse^58,59^; inflicting a form of damage, i.e., a crack, will normally induce a “mobile phase” to the self-healing material, resulting in mending of the two separated surfaces until closure of the crack is achieved. Upon closure, a further immobilisation will take place in which the mechanical properties of the material may be partially or fully restored. In the PBDMS-based proposed materials in this work, the presence of weak B-O crosslink bonds can facilitate this, as these dynamic bonds are constantly in a breaking and reformation reversible process, due to the weak bond energy, thus exhibiting self-healing properties^60–62^. A 6-h timelapse microscopy examination on the nNAMeG with ${\mathrm{X}}_{\mathrm{BA}}^{\mathrm{st}}=0.5$ where an incision was introduced in the middle of the material was performed, to demonstrate its self-healing properties. During the optical examination, intermediate absorption measurements were conducted to study the influence of the incision on the absorption coefficient. Microscopic images in Figs. 8a–e confirmed the capability of the nNAMeG membrane to autonomously self-heal showing the progressive reduction of the damage extent in a time interval of 6 h. More importantly, in order to demonstrate that the healed membranes regain their acoustic properties, absorption coefficient measurements in various time intervals (pristine membrane, fully incised, post 1, 4 and 6 h) have been performed with the transition of the α-coefficient shown in Figs. 8f–j. As anticipated, the absorption curves experienced a radical change as the incision was introduced in the sample, as depicted in Fig. 8g. In agreement with the microscopic imaging, the absorption coefficient peak at 432.3 Hz shows already an increase after 1 h (Fig. 8h), after 4 h (Fig. 8i), and returns to 85% of its initial value after 4 h (Fig. 8i). Finally, the absorption curve reached the initial shape after 6 h, as shown in Fig. 8j. Additional information on the self-healing efficiency of the PBDMS can be found in the Supplementary information.

## Discussion

In conclusion, a novel material is proposed to achieve high absorption coefficient above 0.9 at specific frequencies and high sound transmission loss in the measured low frequency range (500–1000 Hz). A nonlinear frequency shift associated to the stiffening effect was effectively demonstrated in the rheological measurements, led to dynamic shifts of 19.3, 27.1 and 40 Hz for the different inspected stoichiometric BA ratio (i.e. ${\mathrm{X}}_{\mathrm{BA}}^{\mathrm{st}}$) of the absorption frequency peaks. The nonlinear frequency shift was generated by the dynamic transition of the material from a viscous-state to a rubbery-state, where the amount of the energy absorbed to the B-O network for the dynamic cross-link increases. It is also evident that for increased stoichiometric ratios higher values of storage modulus were measured leading to a shift of the absorption peak towards higher frequencies.

The pseudo non-Newtonian material showed an increase in the sound transmission losses up to 35 dB in the measured frequency range (500–1000 Hz). For all the material investigated, the measure STL was consistently above 10 dB. A shift to higher STL values were observed when the sound amplitude was increased with values up to 2 dB. Additional acoustic and microscopy investigation have been conducted to assess the self-healing properties of the nNAMeG and the effect on the acoustic properties. The inspections demonstrated the high capability of the material to self-heal autonomously in a relatively short amount of time, and completely recover its acoustic properties.

These results prove the nNAMeG membranes are able to achieve high and adaptive sound transmission loss in the low frequency range with deep subwavelength thicknesses (up to 1/618 wavelength) in contrast to traditional acoustic materials. These novel self-healable ultrathin materials offer an unprecedented and efficient alternative for noise control application in construction, automotive, aerospace and other engineering applications, where small thicknesses and low-frequency sound transmission loss are in high demand.

## Methods

### Samples preparation

The nNAMeG membranes presented in this work are synthesised from a mixture of Polydimethylsiloxane and Boric Acid with different stoichiometric ratios of Boron to Silicone (0.2, 0.5 and 0.8) at high temperatures (exceeding 150 °C), which results in the creation of a uniform structural network of non-covalent Boron-Oxygen cross bonds that join the different molecular covalent chains. The reagents used were PDMS with an initial viscosity of 200 cSt, Boric Acid and ethanol, without any further purification. All reagents were purchased from Fluorchem UK ltd.

### Microscopy and FT-IR setup

The optical light microscope used for the examination of the surface characteristics and self-healing properties of the samples was a Keyence VHX6000 microscope, equipped with a magnification lens of × 500. The FT-IR instrument employed in the chemical characterisation of the samples was a Perkin Elmer, FT-IR spectrometer.

### Rheology

The rheological measurements for the mechanical characterisation of the nNAMeGs were conducted by means of a TA Instruments DHR-2 rheometer, in a 25 mm parallel plate geometry. The gels were subjected to oscillatory frequency tests, from 0.1 to 100 Hz, with their thicknesses kept constant at 1 mm. During testing, the temperature of the samples was regulated at 20 °C. The resulting storage and loss moduli were compared in logarithmic scales for the whole range of angular frequencies tested (See supplementary material).

### Acoustic measurements

The acoustic investigations were conducted in a two-microphones Kundt tube, with a BMS-2592 Middle loudspeaker positioned at the front of the tube to generate a broadband sweep signal, with two GRAS 40PL 50 Hz-20 kHz, 32-150d B, 10 mV/Pa sensitivity array microphones enclosed to the tube, whereas the inspected samples were placed at the other end of the tube. The apparatus, which consisted of a 50.8 mm aluminium hollow tube has been developed in accordance with the ASTM E 1050‐07 standard^63^, to provide a standing plane wave propagation inside the tube. The loudspeaker is used to send a sweep sinusoidal wave, ranging between the lower and upper frequency limits of the apparatus (i.e., 200–3000 Hz, see Supplementary material). Additionally, the technique utilised to experimentally determine TL consists of a modified two-room setup by means of the above-mentioned Kundt tube, and evaluating sound pressure level (SPL) in the source and anechoic room, with the inspected material being secured in an open window between the two rooms, according with^64^. For the test, 1.1 mm of nNAMeG samples were placed inside 50.8 mm diameter acrylic ring with a 25 mm air gap between the investigated material and the rigid wall on the tube. Detailed information about the membrane-cavity impedance model can be found in the Supplementary information. A 0.18 mm thickness substrate underneath was employed, to provide a consistent constrain to the gel, without affecting the performance of the test samples. For the acoustic tests, four different voltage levels of the sweep sinusoidal wave were used in order to characterise the acoustic behaviour of the proposed gels, as different sound pressure loads were applied (Supplementary Figure 1).

## Supplementary Information

Supplementary Information.

## Data Availability

The authors confirm that the data supporting the findings of this study are available within the article and its Supplementary material. Any further data are available from the authors upon request, please contact corresponding author m.meo@bath.ac.uk.

## References

[1] Yang TL, Chiang D-M, Chen R (2001). Development of a novel porous laminated composite material for high sound absorption. J. Vib. Control.

[2] Küçük M, Korkmaz Y (2012). The effect of physical parameters on sound absorption properties of natural fiber mixed nonwoven composites. Text. Res. J..

[3] Echeverria CA (2019). Engineered hybrid fibre reinforced composites for sound absorption building applications. Resour. Conserv. Recycl..

[4] Yahya MN, Chin DDVS (2017). A review on the potential of natural fibre for sound absorption application. IOP Conf. Series.

[5] Mahzan, S., et al. "Investigation on sound absorption of rice-husk reinforced composite." Proceedings of MUCEET. 19–22 (2099).

[6] Lee J, Swenson GW (1992). Compact sound absorbers for low frequencies. Noise Control Eng. J..

[7] Joshi SV (2004). Are natural fiber composites environmentally superior to glass fiber reinforced composites?. Compos. A Appl. Sci. Manuf..

[8] Khedari J (2004). New low-cost insulation particleboards from mixture of durian peel and coconut coir. Build. Environ..

[9] Sharma GS (2019). Sound absorption by rubber coatings with periodic voids and hard inclusions. Appl. Acoust..

[10] Pieren R (2018). Sound absorption of textile curtains–theoretical models and validations by experiments and simulations. Text. Res. J..

[11] Guzman ADM, Munno MGT (2015). Design of a brick with sound absorption properties based on plastic waste & sawdust. IEEE Access.

[12] Gao W (2001). Energy impacts of recycling disassembly material in residential buildings. Energy Build..

[13] Zhou H (2004). Advances in sound absorption polymers. Progress Chem..

[14] Taşdemir M, Ersoy S, Uluğ E (2012). Effects of HIPS on the sound absorption and impedance ratio of SEBS/HIPS/CaCO3 polymer composites. Polym.-Plast. Technol. Eng..

[15] Cao R (2021). Preparation of natural bio-based Eucommia ulmoides gum/styrene-butadiene rubber composites and the evaluation of their damping and sound absorption properties. Polymer.

[16] Lee J, Kim GH, Ha CS (2012). Sound absorption properties of polyurethane/nano-silica nanocomposite foams. J. Appl. Polym. Sci..

[17] Jiang S (2012). Seven-hole hollow polyester fibers as reinforcement in sound absorption chlorinated polyethylene composites. Appl. Acoust..

[18] Yao K (1995). Acoustic absorption performance of polyacrylic composite latex. J. Appl. Polym. Sci..

[19] Fei Y (2019). Extrusion foaming of lightweight polystyrene composite foams with controllable cellular structure for sound absorption application. Polymers.

[20] Allard J, Atalla N (2009). Propagation of Sound in Porous Media: Modelling Sound Absorbing Materials 2e.

[21] Roger T (2015). Coherent perfect absorption in deeply subwavelength films in the single-photon regime. Nat. Commun..

[22] Wei P (2014). Symmetrical and anti-symmetrical coherent perfect absorption for acoustic waves. Appl. Phys. Lett..

[23] Boccaccio M (2021). Deep-subwavelength-optimized holey-structured metamaterial lens for nonlinear air-coupled ultrasonic imaging. Sensors.

[24] Xiao S (2015). Active control of membrane-type acoustic metamaterial by electric field. Appl. Phys. Lett..

[25] Mei J (2012). Dark acoustic metamaterials as super absorbers for low-frequency sound. Nat. Commun..

[26] Vishnevsky A, Komkin A (2020). Possibilities in finite element simulating of the electroacoustic sound absorber. MATEC Web Conf..

[27] Zhu X (2018). Broadening of the sound absorption bandwidth of the perforated panel using a membrane-type resonator. J. Vib. Acoust..

[28] Tang Y (2017). Deep subwavelength acoustic metamaterial for low-frequency sound absorption. EPL.

[29] Romero-García V (2016). Perfect and broadband acoustic absorption by critically coupled sub-wavelength resonators. Sci. Rep..

[30] Sakagami K (2011). Effect of a honeycomb on the absorption characteristics of double-leaf microperforated panel (MPP) space sound absorbers. Noise Control Eng. J..

[31] Lei L, Zuomin W, Zaixiu J (2011). Effect of sound-absorbing material on a microperforated absorbing construction. Chin. J. Acoust..

[32] Kang J, Brocklesby M (2005). Feasibility of applying micro-perforated absorbers in acoustic window systems. Appl. Acoust..

[33] Rapisarda M, Fierro G-PM, Meo M (2021). Ultralight graphene oxide/polyvinyl alcohol aerogel for broadband and tuneable acoustic properties. Sci. Rep..

[34] Maa D-Y (1998). Potential of microperforated panel absorber. J. Acoust. Soc. Am..

[35] Herdtle, T. *et al.* Effect of Thermal Losses and Fluid-Structure Interaction on the Transfer Impedance of Microperforated Films. *Publications of the Ray W. Herrick Laboratories*. **119**. (2014).

[36] Boccaccio M (2021). Microperforated panel and deep subwavelength Archimedean-inspired spiral cavities for multi-tonal and broadband sound absorption. Appl. Acoust..

[37] Estrada H (2011). Sound transmission through perforated plates with subwavelength hole arrays: A rigid-solid model. Wave Motion.

[38] Liu Z, Picken SJ, Besseling NAM (2014). Polyborosiloxanes (PBSs), synthetic kinetics, and characterization. Macromolecules.

[39] Wu Q (2019). Highly stretchable and self-healing “solid–liquid” elastomer with strain-rate sensing capability. ACS Appl. Mater. Interfaces.

[40] Wang S (2016). Stretchable polyurethane sponge scaffold strengthened shear stiffening polymer and its enhanced safeguarding performance. ACS Appl. Mater. Interfaces.

[41] D’Elia E (2016). Autonomous self-healing structural composites with bio-inspired design. Sci. Rep..

[42] Liang J, Zhang X-H (2015). Rheological properties of SP in shock transmission application. J. Mater. Civ. Eng..

[43] Wang S (2014). Multifunctional polymer composite with excellent shear stiffening performance and magnetorheological effect. J. Mater. Chem. C.

[44] Zhao C (2020). Shear stiffening gels for intelligent anti-impact applications. Cell Rep. Phys. Sci..

[45] Pinto F, Meo M (2017). Design and manufacturing of a novel shear thickening fluid composite (STFC) with enhanced out-of-plane properties and damage suppression. Appl. Compos. Mater..

[46] Myronidis, K. *et al*. A novel, bioinspired, non-Newtonian energy absorption medium for the protection of composite laminates under low velocity impact (LVI). In *Proc. of SPIE*, Vol. 2022.

[47] Myronidis K (2022). Polyborosiloxane-based, dynamic shear stiffening multilayer coating for the protection of composite laminates under low velocity impact. Compos. Sci. Technol..

[48] Efimenko K, Wallace WE, Genzer J (2002). Surface modification of Sylgard-184 Poly(dimethyl siloxane) networks by ultraviolet and ultraviolet/ozone treatment. J. Colloid Interface Sci..

[49] Khorasani MT, Mirzadeh H, Kermani Z (2005). Wettability of porous polydimethylsiloxane surface: Morphology study. Appl. Surf. Sci..

[50] Mata A, Fleischman AJ, Roy S (2005). Characterization of polydimethylsiloxane (PDMS) properties for biomedical micro/nanosystems. Biomed. Microdevice.

[51] Drozdov FV (2019). Crosslinked polymers based on polyborosiloxanes: Synthesis and properties. J. Organomet. Chem..

[52] Zinchenko GA, Mileshkevich VP, Kozlova NV (1981). Investigation of the synthesis and hydrolytic degradation of polyborodimethylsiloxanes. Polym. Sci. U. S. S. R..

[53] Barnes HA, Hutton JF, Walters K (1989). An Introduction to Rheology.

[54] Gutierrez-Lemini D, Viscoelasticity E (2014). Springer Science + Business Media: Arlington.

[55] Shravage P, de’Sa K (2009). Effect of macroscopic parameters on sound absorption and sound transmission loss of porous materials. J. Acoust. Soc. Am..

[56] Oldham DJ, Egan CA, Cookson RD (2011). Sustainable acoustic absorbers from the biomass. Appl. Acoust..

[57] Ren S (2017). A semi-analytical model for the influence of temperature on sound propagation in sintered metal fiber materials. Mater. Des..

[58] Hager MD (2010). Self-healing materials. Adv. Mater..

[59] Hia IL, Vahedi V, Pasbakhsh P (2016). Self-healing polymer composites: prospects, challenges and applications. Polym. Rev..

[60] Tang F (2022). Protective performance and dynamic behavior of composite body armor with shear stiffening gel as buffer material under ballistic impact. Compos. Sci. Technol..

[61] Wang S (2015). Rate-dependent and self-healing conductive shear stiffening nanocomposite: A novel safe-guarding material with force sensitivity. J. Mater. Chem. A.

[62] Wu J, Cai LH, Weitz DA (2017). Tough self-healing elastomers by molecular enforced integration of covalent and reversible networks. Adv. Mater..

[63] Testing, A.S.f. and Materials, *ASTM E 1050‐07. Standard Test Method for Impedance and Absorption of Acoustical Materials Using a Tube, Two Microphones and a Digital Frequency Analysis System*. (2007).

[64] Barnard, A.R. and M.D. Rao, Measurement of sound transmission loss using a modified four microphone impedance tube. *Proceedings of the ASME Noise Control and Acoustics Division (Noise-Con’04)* (2004).

