# Supplementary material for: A multifunctional ultra-thin acoustic membrane with self-healing properties for adaptive low-frequency noise control
Source: Sci Rep. 2022 Oct 22;12:17790. doi: 10.1038/s41598-022-22441-4 (PMC9588055; doi:10.1038/s41598-022-22441-4)
Supplement: Supplementary file 1 — Supplementary Information. [file 41598_2022_22441_MOESM1_ESM.docx]

A multifunctional ultra-thin acoustic membrane with self-healing properties for adaptive low frequency noise control .

Marco Boccaccio^1^, Konstantinos Myronidis^1^, Michael Thielke^1^, Michele Meo^1^, Fulvio Pinto^1^

^1^University of Bath, Department of Mechanical Engineering, Bath, BA27AY, UK

**Shear Stiffening Mechanism**

The shear stiffening mechanism of the SSGs relies on the dynamic boron-siloxane crosslinking ligands. As illustrated in Supplementary Figure 1, weak electron intermolecular interactions between the vacant 2-p orbitals of the boron atom and the free pair of electrons in the oxygen atoms of the siloxane can be assembled [1-3]; the stiffening behaviour of SSGs is based on the dynamic breaking and recovering behaviour of this B-O bond [4].

| 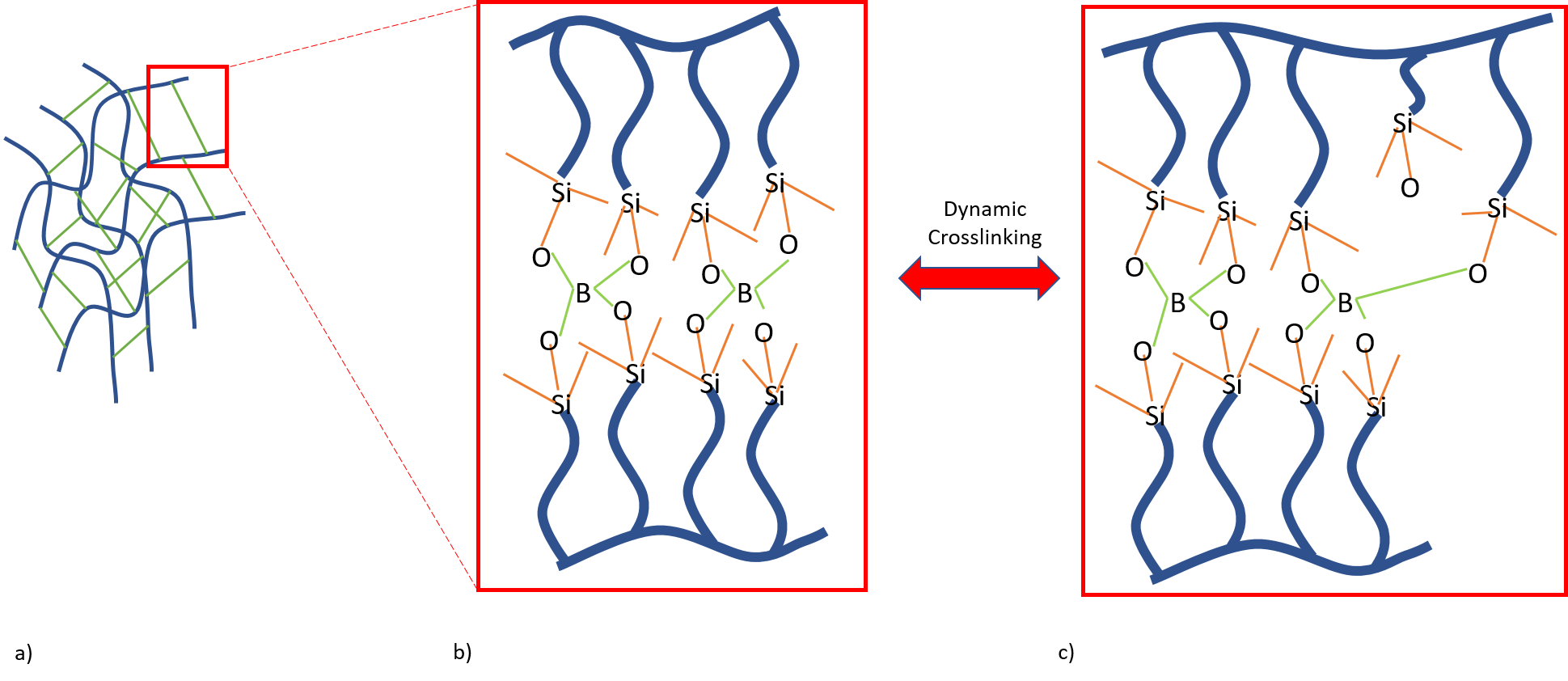  Supplementary Figure 1 Illustration of crosslinking process of boron-oxygen dynamic bonds, a) network overall view with blue lines representing polymeric chains and green lines the dynamic bond, b) focusing on the B-O bonds and c) breakage and recovering of the bonds |
| --- |

An external loading induced on SSGs at low strain rate will macroscopically result in a rather soft material, with increased flowability, compared to its initial state; conversely, when the strain rate of the external load increases, this will give rise to the material’s stiffness [5]. Microscopically, at low strain rates, the network of supramolecular bonds has sufficient time to break and the polymeric chains disentangle and deform, as the characteristic breakage time of the B-O bond is a lot shorter than that of the molecular chains, allowing a certain internal mobility. In these strain rates, SSGs are at a viscous liquid state and can be easily deformed [6]. At higher strain rates though, the B-O crosslinks do not have sufficient time to be deconstructed, creating greater resistance against the disentanglement of the polymeric chains, with the rotation of these controlling the mechanical properties of the SSGs, as these show an inability to readjust to dynamic loading conditions [7, 8]. In these strain rates, the SSGs will exhibit their stiffening behaviour, dynamically transitioning from the viscous state demonstrated at low strain rates, to a rubbery state [5]. This dynamic phase transition of SSGs, from viscous to rubbery state, between low and intermediate strain rates, is responsible for their excellent energy absorbing characteristics [9, 10]. At high strain rates (i.e. >4000 S^-1^), SSGs undergo another phase transition, from rubbery to glassy state, however at this range the loading rate is at an adequate level for the B-O bonds to be broken, with the polymeric chains at this phase organised in groups and appearing as a crystal lattice [11]. The rate-dependent response and dynamic transition between phases of SSGs is illustrated in Supplementary Figure 2. At low strain rates, with the material at the viscous state, the polymeric chains disentangle and stretch, at intermediate rates the material exhibits a phase transition to rubbery state and the stiffening mechanism is triggered with molecular chains remaining in place and B-O bonds deconstructed and reformed. Finally, at high strain rates the polymeric network appears as crystal lattice, with the molecular chains organised as a group.

| 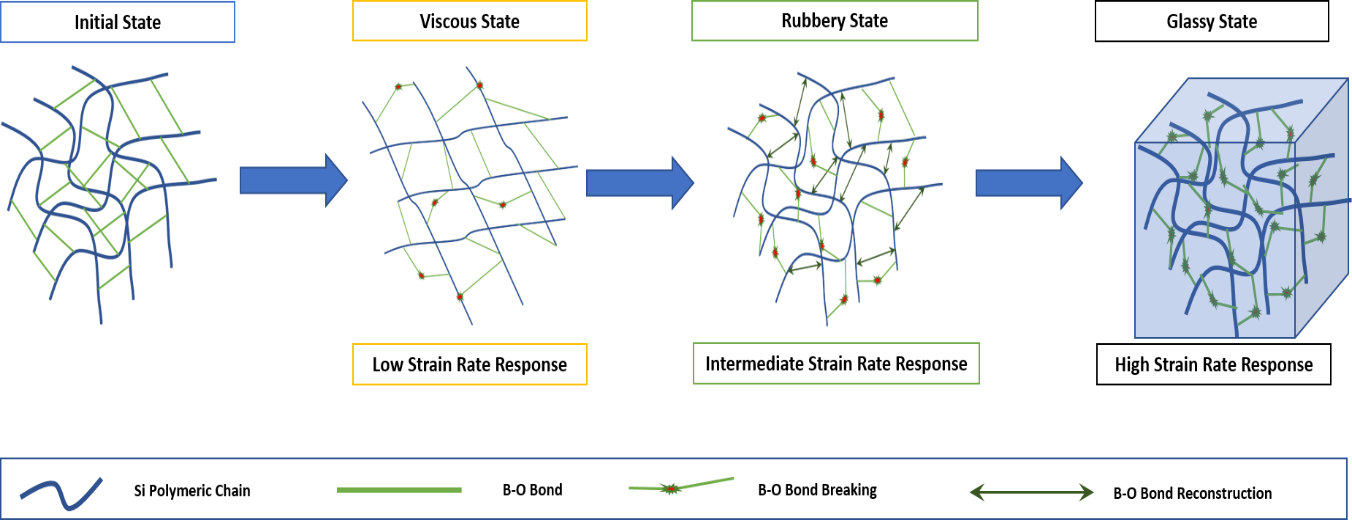  Supplementary Figure 2 Strain-dependent stiffening mechanism of SSGs and dynamic phase transition. |
| --- |

In addition to the intrinsic mechanical properties, the dynamic crosslinking of the B-O bond attributes to self-healing characteristics of the gels, as the close proximity of the boron and oxygen atoms enables them to be recombined in the vicinity of an external load [12].

**Rheology and Mechanical Characterisation of SSG**

It is apparent from the underlying mechanism of the SSGs that in the microscopic view of the structure, flow and deformation occurs and that their mechanical properties are rate dependent [9]. An interrelation between the applied load, the deformation occurred and time, can be provided by means of rheological measurements [13]. In particular, oscillatory rheological measurements yield information regarding the material’s resistance to deformation over a range of time scales. The output is provided by means of dynamic storage (G’) and loss (G’’) moduli (Pa), over a range of angular frequencies (Hz), in response to an applied sinusoidal shear strain [13, 14]. The elastic behaviour of the material is denoted by G’, while their viscous behaviour by G’’[3, 15]. A material exhibiting G’>G’’, denotes prevalence of elastic properties and a solid-like behaviour; on the contrary, when G’<G’’ is satisfied the material exhibits a more liquid-like behaviour and energy loss in the form of heat dissipation [15-17]. At the critical point where these moduli intersect (G’=G’’), a phase transition in the material occurs from viscous to rubbery state, with the appearance of the critical point being correlated to trapped entanglements appearing to the material’s network, due to the kinematic viscosity of the precursor employed [18, 19]. At low strain rates and up to G’=G’’, SSGs are in a viscous phase which is reflected in the predominance of the G’’, whereas past this critical point SSGs enter a diffuse transition to a rubbery state, with G’ becoming predominant. The relationship between rheological measurements and phase transition in SSGs is illustrated in Supplementary Figure 3.

| 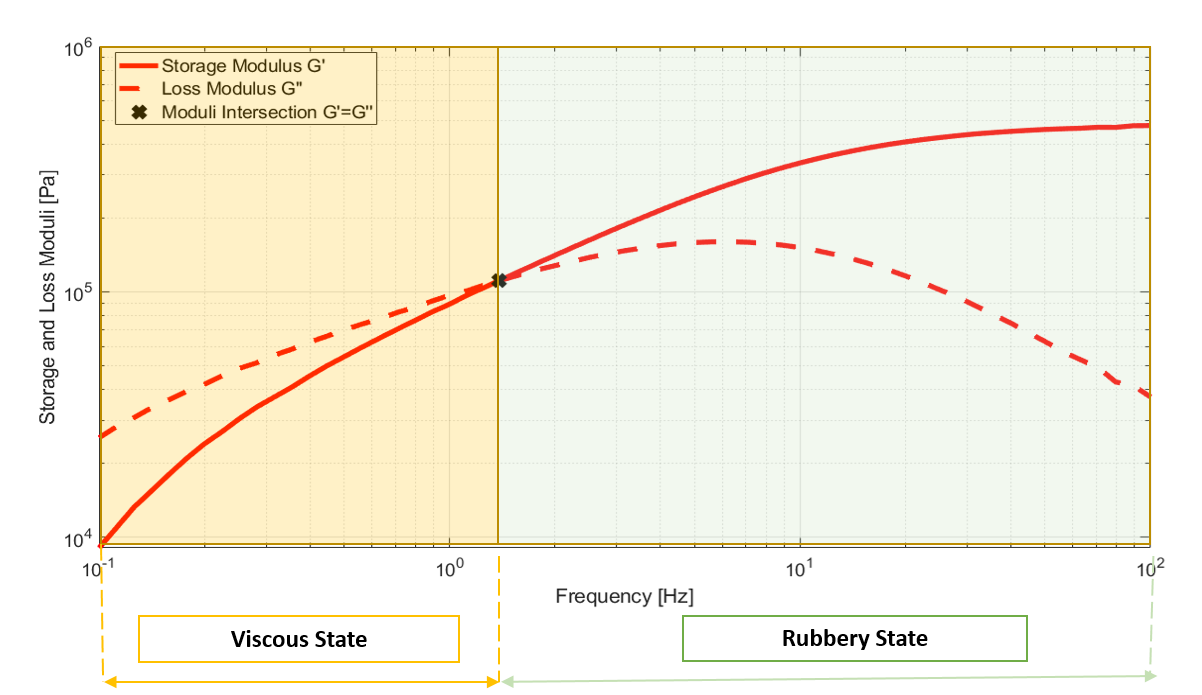  Supplementary Figure 3 Relationship between rheological measurements and phase transition in SSGs. |
| --- |

In summary, at low strain rates SSGs are in a viscous state and very deformable; in this range of angular frequencies, G’’ records higher values than G’ and the material dissipates energy in the form of heat loss. At a critical point where G’’ and G’ intersect, a phase transition is initiated and the material enters a rubbery state with a solid-like appearance; in this range G’ values are predominant signifying the material’s ability to store more energy. This dynamic phase transition of the SSGs is accountable for the excellent energy absorption characteristics.

**Oscillation Rheology Theory**

Following the work of Rogers [20] and deploying Boltzmann’s superposition principle, supplementary equation (SE) 1 follows, where σ indicates the applied stress at present time t, G is the relaxation modulus and $\dot{\gamma}$ the strain rate and $s=t-t^{'}.$

|  | $\boldsymbol{\sigma=}\int_{\boldsymbol{-\infty}}^{\mathbf{t}} \mathbf{G}\left( \mathbf{t-}\mathbf{t}^{\mathbf{'}} \right)\dot{\boldsymbol{\gamma}}\left( \mathbf{t}^{\mathbf{'}} \right)\mathbf{d}\mathbf{t}^{\mathbf{'}}\mathbf{=}\int_{\mathbf{0}}^{\boldsymbol{\infty}} \mathbf{G}\left( \mathbf{s} \right)\dot{\boldsymbol{\gamma}}\left( \mathbf{t-s} \right)\mathbf{ds}$ | SE 1 |
| --- | --- | --- |

Applying a sinusoidal strain with an amplitude of $\gamma_{0}$ and angular frequency ω on a viscoelastic material during oscillatory measurements, a cosinusoidal rate of strain is also applied to it, transforming SE 1 to *SE 2* .

| $\boldsymbol{\sigma=}\int_{\boldsymbol{0}}^{\boldsymbol{\infty}} \boldsymbol{G(s)}\boldsymbol{\gamma}_{\boldsymbol{0}}\boldsymbol{\omega}\cos\left[ \boldsymbol{\omega}\left( \boldsymbol{t-s} \right) \right]\boldsymbol{ds=}\boldsymbol{\gamma}_{\boldsymbol{0}}\left[ \boldsymbol{\omega}\int_{\boldsymbol{0}}^{\boldsymbol{\infty}} \boldsymbol{G(s)}\sin\left( \boldsymbol{\omega}\boldsymbol{s} \right)\boldsymbol{ds} \right]\sin\boldsymbol{\omega}\boldsymbol{t}\boldsymbol{+}\boldsymbol{\gamma}_{\boldsymbol{0}}\left[ \boldsymbol{\omega}\int_{\boldsymbol{0}}^{\boldsymbol{\infty}} \boldsymbol{G(s)}\cos\left( \boldsymbol{\omega}\boldsymbol{s} \right)\boldsymbol{ds}\cos\boldsymbol{(}\boldsymbol{\omega}\boldsymbol{t)} \right]$ | **SE 2** |
| --- | --- |

Since the terms in the brackets are functions of the frequency only, *SE 2* reduces to SE 3.

|  | $\boldsymbol{\sigma=}\boldsymbol{\gamma}_{\mathbf{0}}\mathbf{(}\mathbf{G}^{\mathbf{'}}\left( \boldsymbol{\omega} \right)\sin\left( \boldsymbol{\omega t} \right)\mathbf{+}\mathbf{G}^{\mathbf{''}}\left( \boldsymbol{\omega} \right)\cos\left( \boldsymbol{\omega t} \right)$ | SE 3 |
| --- | --- | --- |

In an ideal elastic material, where there is a linear relationship between stress and strain and the occurrence of maximum stress coincides that of maximum strain, both terms will be in phase; on the contrary, for an ideal viscous material, where the maximum stress occurs at the maximum value of the rate of deformation, the stress and strain terms are out of phase by $\frac{\pi}{2}$ radians [15]. In viscoelastic materials, such as the SSGs, this phase transition appears between these extremes. This phase difference, namely δ, determines how the viscous and elastic properties of the materials contribute to the overall stiffness, the complex modulus (G*) [21]. Trigonometry can be employed to express the relationship between dynamic moduli and δ, or making use of complex notation, since G* is the Complex modulus. Both relationships are illustrated in Supplementary Figure 4 below. In complex representation, G’ is the real part whereas G’’ is the imaginary part, and the summation of these (*SE 4*) yields G*, with $\dot{i}$ being the imaginary unit [22].

|  | $\mathbf{G}^{\mathbf{*}}\mathbf{=}\mathbf{G}^{\mathbf{'}}\mathbf{+}\mathbf{iG}^{\mathbf{''}}$ | SE 4 |
| --- | --- | --- |


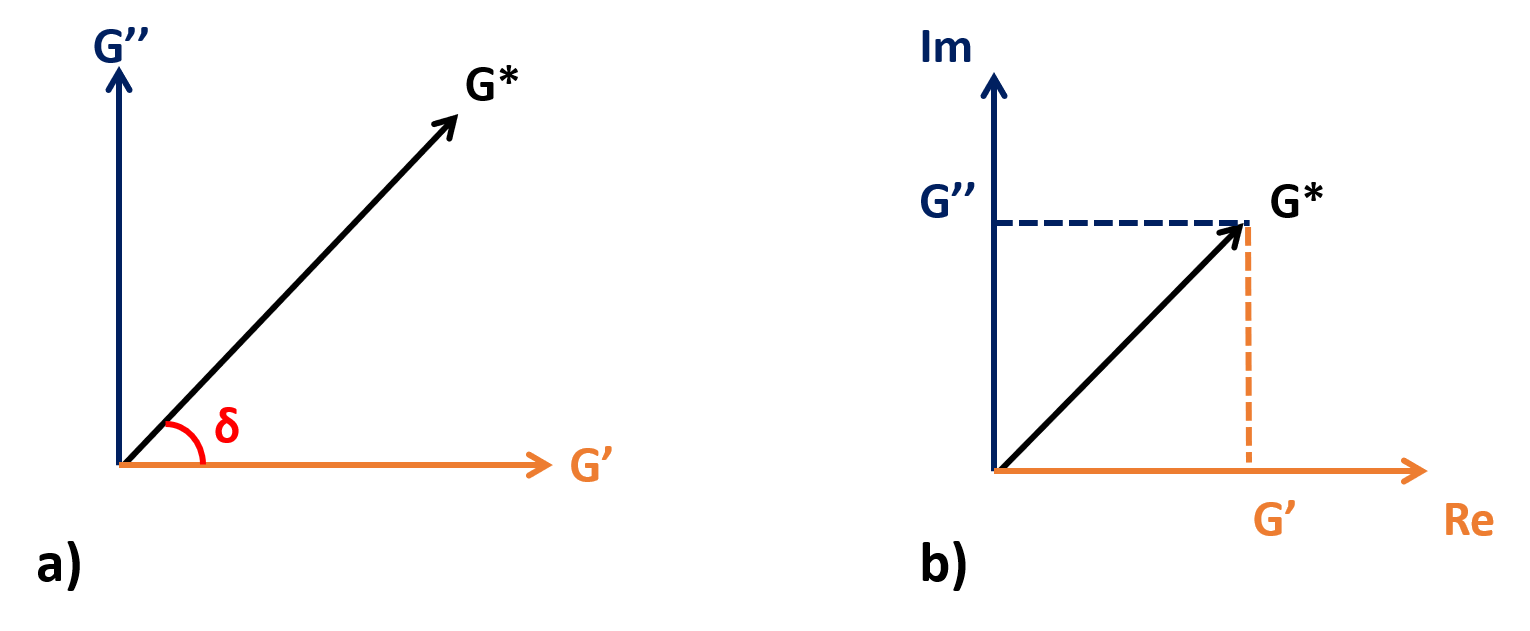


Supplementary Figure 4 Relationship between Complex (G*), Storage (G') and Loss (G'') moduli, a) employing trigonometry and phase shift δ and b) on a complex plane (Argand diagram).

**Transfer function Method for absorption measurements**

Sound absorption represents the measure of the quantity of energy removed from a sound wave as it passes through the material being inspected. These mechanisms can be analytically described and measured by analysing the acoustic impedance of the system and via the Transfer Function Method (TFM). Generally, propagation of an oscillation expressed in terms of potential $\phi$ at a fixed speed can be described by a linear second-order partial differential equation [23]:

|  | $\boldsymbol{\nabla}^{\mathbf{2}}\boldsymbol{\phi=\rho}\frac{\boldsymbol{\partial}^{\mathbf{2}}\boldsymbol{\phi}}{\boldsymbol{\partial}\mathbf{t}^{\mathbf{2}}}$ | SE 5 |
| --- | --- | --- |

Where $\rho$ denotes the fluid density, and t is the time variable. A displacement solution of SE 5 in a compressible and lossless fluid is given by:

|  | $\boldsymbol{\phi}\left( \mathbf{x,t} \right)\mathbf{=}\frac{\mathbf{A}}{\boldsymbol{\rho}\boldsymbol{\omega}^{\mathbf{2}}}$ | SE 6 |
| --- | --- | --- |

Where $\omega$ is the angular frequency, $k=\omega\sqrt{\rho/K}$ is the wave number, K is the bulk modulus of the fluid and A represents the amplitude of the sound pressure $p\left( x,t \right)=Aexp[j\left( \omega t-kx \right)]$. In the assumption of unidirectional propagation (i.e.$u_{y}=u_{z}=0$), the displacement vector component u can be expressed as follows:

|  | $\mathbf{u}_{\mathbf{x}}\left( \mathbf{x,t} \right)\mathbf{=}{\frac{\mathbf{-jkA}}{\boldsymbol{\rho}\boldsymbol{\omega}^{\mathbf{2}}}\boldsymbol{exp[j\omega}\left( \mathbf{t-}\mathbf{x}/\mathbf{c} \right)\mathbf{]}}$ | SE 7 |
| --- | --- | --- |

Where c denotes the sound speed within the fluid. SEs 6-7 can be used to describe propagation of the harmonic plane wave travelling along the x-direction. With these regards, pressure and velocity are related as follow:

|  | $\mathbf{v}_{\mathbf{x}}\left( \mathbf{x,t} \right)\mathbf{=}{\frac{\mathbf{1}}{\mathbf{Z}}\mathbf{p}\left( \mathbf{x,t} \right)}$ | SE 8 |
| --- | --- | --- |

Where Z is the complex acoustic impedance of the system and represents a measure of the resistance encountered by an ultrasound beam passing through the system. The complex acoustic impedance can be evaluated via transfer function methods, by decomposing a standing wave in its reflected and incident components, by means of a transfer function relation between sound pressure amplitude measured in two different locations, as illustrated in Supplementary Figure 5.

| 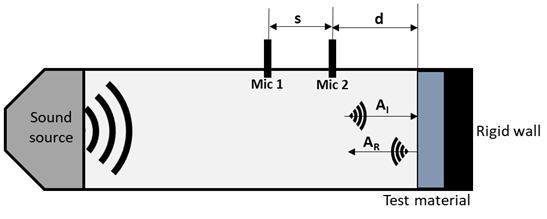  Supplementary Figure 5 Two microphones impedance tube testing. |
| --- |

The complex sound pressure captured by the microphones 1 and 2 can be evaluated as follows:

|  | $\mathbf{P}_{\mathbf{1}}\mathbf{=}\mathbf{A}_{\mathbf{I}}\exp\mathbf{[jk}\left( \mathbf{d+s} \right)\mathbf{]}\mathbf{+}\mathbf{A}_{\mathbf{R}}\mathbf{exp[jk}\left( \mathbf{d+s} \right)\boldsymbol{]}$ | SE 9 |
| --- | --- | --- |
|  | $\mathbf{P}_{\mathbf{2}}\mathbf{=}\mathbf{A}_{\mathbf{I}}\exp\left( \mathbf{jk}\mathbf{d} \right)\mathbf{+}\mathbf{A}_{\mathbf{R}}\mathbf{exp(jk}\mathbf{d}\mathbf{)}$ | **SE 10** |

Where A­*­*_I_ and A_R_ are the amplitudes of the incident and reflected wave respectively, **s** is the spacing between the two microphones and **d** represents the distance between the test material and the closed microphone. The reflection coefficient R can be evaluated trough the transfer function between the two microphones signals H_1,2_,and the transfer function of the incident wave H_i_ and that of the reflected wave H_R_, as follows[24]:

|  | $\mathbf{R=}\frac{\mathbf{H}_{\mathbf{1,2}}\mathbf{-}\mathbf{H}_{\mathbf{i}}}{\mathbf{H}_{\mathbf{R}}\mathbf{-}\mathbf{H}_{\mathbf{12}}}\mathbf{exp[2jk}\left( \mathbf{d+s} \right)\mathbf{]}$ | SE 11 |
| --- | --- | --- |

According to SE 11, the complex acoustic impedance Z on the cross-sectional area of the tube at a distance d can be obtained as:

|  | $\frac{\mathbf{Z}}{\boldsymbol{\rho c}}\mathbf{=}\frac{\mathbf{1+R}}{\mathbf{1-R}}\mathbf{=j}\frac{\mathbf{H}_{\mathbf{12}}\sin\left( \mathbf{kd} \right)\mathbf{-sin[k}\left( \mathbf{d-s} \right)\mathbf{]}}{\mathbf{cos[k}\left( \mathbf{d-s} \right)\mathbf{-}\mathbf{H}_{\mathbf{12}}\mathbf{cos(kd)}}$ | SE 12 |
| --- | --- | --- |

The sound absorption coefficient $\alpha$ of a material represents the ratio of the total sound energy which is absorbed by the tested material. Thus using the reflection coefficient R in SEs 11-12 can be expressed as [25, 26]:

|  | $\boldsymbol{\alpha=1-}\left\vert\mathbf{R} \right\vert^{\mathbf{2}}\boldsymbol{=}\frac{\mathbf{H}_{\mathbf{R}}^{\mathbf{2}}\mathbf{-}\mathbf{H}_{\mathbf{I}}^{\mathbf{2}}\mathbf{-2}\mathbf{H}_{\mathbf{12}}\mathbf{(}\mathbf{H}_{\mathbf{R}}\mathbf{-}\mathbf{H}_{\mathbf{I}}\mathbf{)}}{\left( \mathbf{H}_{\mathbf{R}}\mathbf{-}\mathbf{H}_{\mathbf{12}} \right)^{\mathbf{2}}}$ | SE 13 |
| --- | --- | --- |

The test apparatus shown in Supplementary Figure 5 was has been designed in accordance with ASTM E-1050 [27] for $\boldsymbol{\alpha}$-coefficient A straight 15 mm thickness aluminium tube with an internal diameter of 50.8 mm has been used. With this thickness, the tube wall can be assumed as acoustically rigid. The working frequency range of the apparatus is:

|  | $\mathbf{f}_{\boldsymbol{l}}\mathbf{<f<}\mathbf{f}_{\mathbf{u}}$ | SE 14 |
| --- | --- | --- |

With $\mathbf{f}_{\boldsymbol{l}}$ **_­­_**and $\mathbf{f}_{\mathbf{u}}$ being the lower and frequency limit. The upper frequency limit depends on the diameter of the tube D the speed of sound c_0_, a parameter K which is function of the tube cross-section (K = 0.586 for circular section) and the microphone spacing s, in order to guarantee plane wave propagation:

|  | $\mathbf{f}_{\mathbf{u}}\mathbf{=}\frac{\mathbf{K}\mathbf{c}_{\mathbf{0}}}{\mathbf{d}}$ | SE 15 |
| --- | --- | --- |

Additionally, the maximum microphone spacing may not exceed the 80% of the shortest half wavelength (i.e., upper frequency):

|  | $\mathbf{f}_{\mathbf{u}}\mathbf{>}\frac{\boldsymbol{0.4}\mathbf{c}_{\mathbf{0}}}{\mathbf{s}}$ | SE 16 |
| --- | --- | --- |

The lower frequency limit mainly depends on the microphone spacing. With this regard, the minimum microphone spacing may exceed 1% of the wavelength corresponding to the lower frequency, as follows:

|  | $\mathbf{f}_{\mathbf{l}}\mathbf{>}\frac{\mathbf{c}}{\mathbf{100s}}$ | SE 17 |
| --- | --- | --- |

Supplementary Table 1 denotes the test rig geometrical parameter and the respective frequency range for absorption measurement, according with SEs 14-17 and the respective standard test methods.

Supplementary Table 1 Test apparatus geometrical parameters for sound absorption measurements.

| Frequency Range | 150 – 1000 Hz |
| --- | --- |
| Diameter | 50.8 mm |
| Distance loudspeaker – Sample | 330 mm |
| Distance Mic 1 – Mic 2 | 22.5 mm |
| Distance Mic 2 - Sample | 140 mm |

**Modified four microphones impedance tube for Sound Transmission Loss measurement**

The test apparatus used to perform Sound transmission loss consists of a modified version of the standard two microphones impedance tube in [27], where two additional microphones were placed at the back side of the test material [28-30], as seen in Supplementary Figure 6 below.


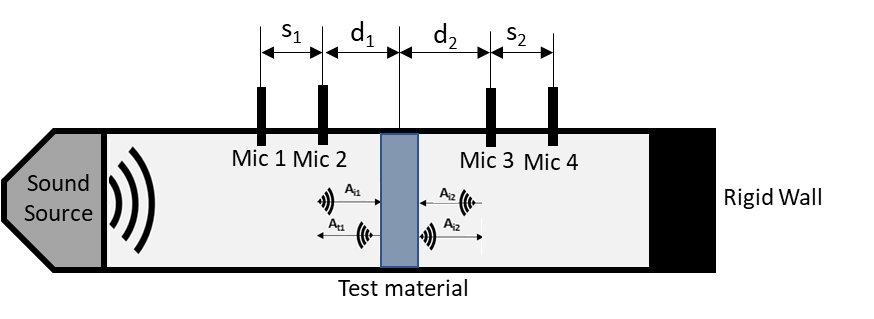


Supplementary Figure 6 Four microphone modified impedance tube.

As shown in Supplementary Figure 6, the sound field inside tube is composed of the incident and transmitted component of the forward travelling wave, A­_i1_  and A_t2_ , and the incident and transmitted component of the backward travelling wave, A_i2_ and A_t1_. The measured complex sound pressure at the four microphones location can be evaluated as follows[28]:

|  | $\left\{ \begin{aligned} \boldsymbol{P}_{\boldsymbol{1}}\boldsymbol{=}\boldsymbol{A}_{\boldsymbol{i}\boldsymbol{1}}\boldsymbol{e}^{\boldsymbol{j[\omega t-k}\left( \boldsymbol{s}_{\boldsymbol{1}}\boldsymbol{+}\boldsymbol{d}_{\boldsymbol{1}} \right)\boldsymbol{]}}\boldsymbol{+}\boldsymbol{A}_{\boldsymbol{t}\boldsymbol{1}}\boldsymbol{e}^{\boldsymbol{j[\omega t-k}\left( \boldsymbol{s}_{\boldsymbol{1}}\boldsymbol{+}\boldsymbol{d}_{\boldsymbol{1}} \right)\boldsymbol{]}} \\ \boldsymbol{P}_{\boldsymbol{2}}\boldsymbol{=}\boldsymbol{A}_{\boldsymbol{i}\boldsymbol{1}}\boldsymbol{e}^{\boldsymbol{j[\omega t-k}\left( \boldsymbol{d}_{\boldsymbol{1}} \right)\boldsymbol{]}}\boldsymbol{+}\boldsymbol{A}_{\boldsymbol{t}\boldsymbol{1}}\boldsymbol{e}^{\boldsymbol{j[\omega t-k}\left( \boldsymbol{d}_{\boldsymbol{1}} \right)\boldsymbol{]}} \\ \boldsymbol{P}_{\boldsymbol{3}}\boldsymbol{=}\boldsymbol{A}_{\boldsymbol{t}\boldsymbol{2}}\boldsymbol{e}^{\boldsymbol{j[\omega t-k}\left( \boldsymbol{d}_{\boldsymbol{2}} \right)\boldsymbol{]}}\boldsymbol{+}\boldsymbol{A}_{\boldsymbol{i}\boldsymbol{2}}\boldsymbol{e}^{\boldsymbol{j[\omega t-k}\left( \boldsymbol{d}_{\boldsymbol{2}} \right)\boldsymbol{]}} \\ \boldsymbol{P}_{\boldsymbol{4}}\boldsymbol{=}\boldsymbol{A}_{\boldsymbol{i}\boldsymbol{1}}\boldsymbol{e}^{\boldsymbol{j[\omega t-k}\left( \boldsymbol{s}_{\boldsymbol{2}}\boldsymbol{+}\boldsymbol{d}_{\boldsymbol{2}} \right)\boldsymbol{]}}\boldsymbol{+}\boldsymbol{A}_{\boldsymbol{t}\boldsymbol{2}}\boldsymbol{e}^{\boldsymbol{j[\omega t-k}\left( \boldsymbol{s}_{\boldsymbol{2}}\boldsymbol{+}\boldsymbol{d}_{\boldsymbol{2}} \right)\boldsymbol{]}} \end{aligned} \right.$ | SE 18 |
| --- | --- | --- |

Where the distances s_1_, d_­1_, s_2_, d_2_ are illustrated in Figure. The amplitudes can be evaluated by rewriting *SE 18* as follows:

|  | $\left\{ \begin{aligned} \mathbf{A}_{\mathbf{i1}}\mathbf{=}\frac{\mathbf{j(}\mathbf{P}_{\mathbf{1}}\mathbf{e}^{\mathbf{jkd1}}\mathbf{-}\mathbf{P}_{\mathbf{2}}\mathbf{e}^{\mathbf{jk}\left( \mathbf{s}_{\mathbf{1}}\mathbf{+}\mathbf{d}_{\mathbf{1}} \right)}}{\mathbf{2 sin k}\mathbf{s}_{\mathbf{1}}} \\ \mathbf{A}_{\mathbf{t1}}\mathbf{=}\frac{\mathbf{j(}\mathbf{P}_{\mathbf{2}}\mathbf{e}^{\mathbf{-jk}\left( \mathbf{s}_{\mathbf{1}}\mathbf{+}\mathbf{d}_{\mathbf{1}} \right)}\mathbf{-}\mathbf{P}_{\mathbf{1}}\mathbf{e}^{\mathbf{-jk}\mathbf{d}_{\mathbf{1}}}}{\mathbf{2 sin k}\mathbf{s}_{\mathbf{1}}} \\ \mathbf{A}_{\mathbf{t2}}\mathbf{=}\frac{\mathbf{j(}\mathbf{P}_{\mathbf{3}}\mathbf{e}^{\mathbf{jk}\left( \mathbf{s}_{\mathbf{2}}\mathbf{+}\mathbf{d}_{\mathbf{2}} \right)}\mathbf{-}\mathbf{P}_{\mathbf{4}}\mathbf{e}^{\mathbf{jk}\left( \mathbf{d}_{\mathbf{2}} \right)}}{\mathbf{2 sin k}\mathbf{s}_{\mathbf{2}}} \\ \mathbf{A}_{\mathbf{i2}}\mathbf{=}\frac{\mathbf{j(}\mathbf{P}_{\mathbf{1}}\mathbf{e}^{\mathbf{jk}\mathbf{d}_{\mathbf{2}}}\mathbf{-}\mathbf{P}_{\mathbf{2}}\mathbf{e}^{\mathbf{jk}\left( \mathbf{s}_{\mathbf{2}}\mathbf{+}\mathbf{d}_{\mathbf{2}} \right)}}{\mathbf{2 sin k}\mathbf{s}_{\mathbf{2}}} \end{aligned} \right.$ | SE 19 |
| --- | --- | --- |

The basic theory relies on the definition of the transmission loss matrix, which represents an intrinsic property of the test material, and relates the forward and backward propagating wave, as follows:

|  | $\binom{\mathbf{A}_{\mathbf{i1}}}{\mathbf{A}_{\mathbf{t1}}}\mathbf{=}\left[ \begin{matrix} \mathbf{X}_{\boldsymbol{\alpha}}\boldsymbol{(\omega)} & \mathbf{X}_{\boldsymbol{\beta}}\boldsymbol{(\omega)} \\ \mathbf{X}_{\boldsymbol{\gamma}}\boldsymbol{(\omega)} & \mathbf{X}_{\boldsymbol{\delta}}\boldsymbol{(\omega)} \end{matrix} \right]\mathbf{=}\binom{\mathbf{A}_{\mathbf{t2}}}{\mathbf{A}_{\mathbf{i2}}}$ | SE 20 |
| --- | --- | --- |

Where the coefficient of interest is the transmission loss coefficient $X_{\alpha}(\omega)$, which can be evaluated via determinant method. Consequently, Sound Transmission Loss is defined as the transmission loss coefficient expressed in dB, as:

|  | $\mathbf{TL=10 log}{}_{\mathbf{10}}\left( \frac{\boldsymbol{1}}{\boldsymbol{X}_{\boldsymbol{\alpha}}\boldsymbol{(\omega)}} \right)$ | SE 21 |
| --- | --- | --- |

STL measurements were performed between frequencies of 500-3000 Hz with the settings found in Supplementary Table 2 below.

Supplementary Table 2 Test apparatus geometrical parameters for sound transmission loss measurements.

| Frequency Range | 500 – 3000 Hz |
| --- | --- |
| Diameter | 50.8 mm |
| Distance loudspeaker – Sample | 330 mm |
| Distance Mic 1 – Mic 2 | 22.5 mm |
| Distance Mic 3 – Mic 4 | 22.5 mm |
| Distance Mic 2 – Sample | 140 mm |
| Distance Sample – Mic 3 | 140 mm |

**Self-Healing Efficiency**

In order to highlight the material’s self-healing efficiency on this matter, ***SE 22*** was introduced:

| $SHE = \frac{a_{incision}}{a_{intact}} \times100\%$ |  | **SE 22** |
| --- | --- | --- |

Where SHE is the Self-Healing Efficiency in percentage, α_incision_ represents the absorption coefficient evaluated on the sample at any given time and α_intact_ the absorption coefficient of the pristine sample.

Supplementary Table 3 Self-Healing Efficiency of PBDMS

| Time Interval [hours] | α Coefficient Peaks | Self-Healing Efficiency [%] |
| --- | --- | --- |
| Pristine | 0.9 | - |
| 0 (initial cut) | 0.41 | 45.5 |
| 1 | 0.53 | 58.8 |
| 4 | 0.8 | 88.8 |
| 6 | 0.9 | 100 |

Based on the value shown in the Supplementary Table 3, the SHE parameters give a clearer understanding on the fact that the incision does not affect the acoustic performance of the proposed materials, once been healed.

Since the polymeric nature of the material is susceptible to changes in environmental conditions, further tests were carried out (incision-induced tests) to evaluate the self-healing performance of the membrane under different conditions as can be seen in Supplementary Figure 7 below.

| 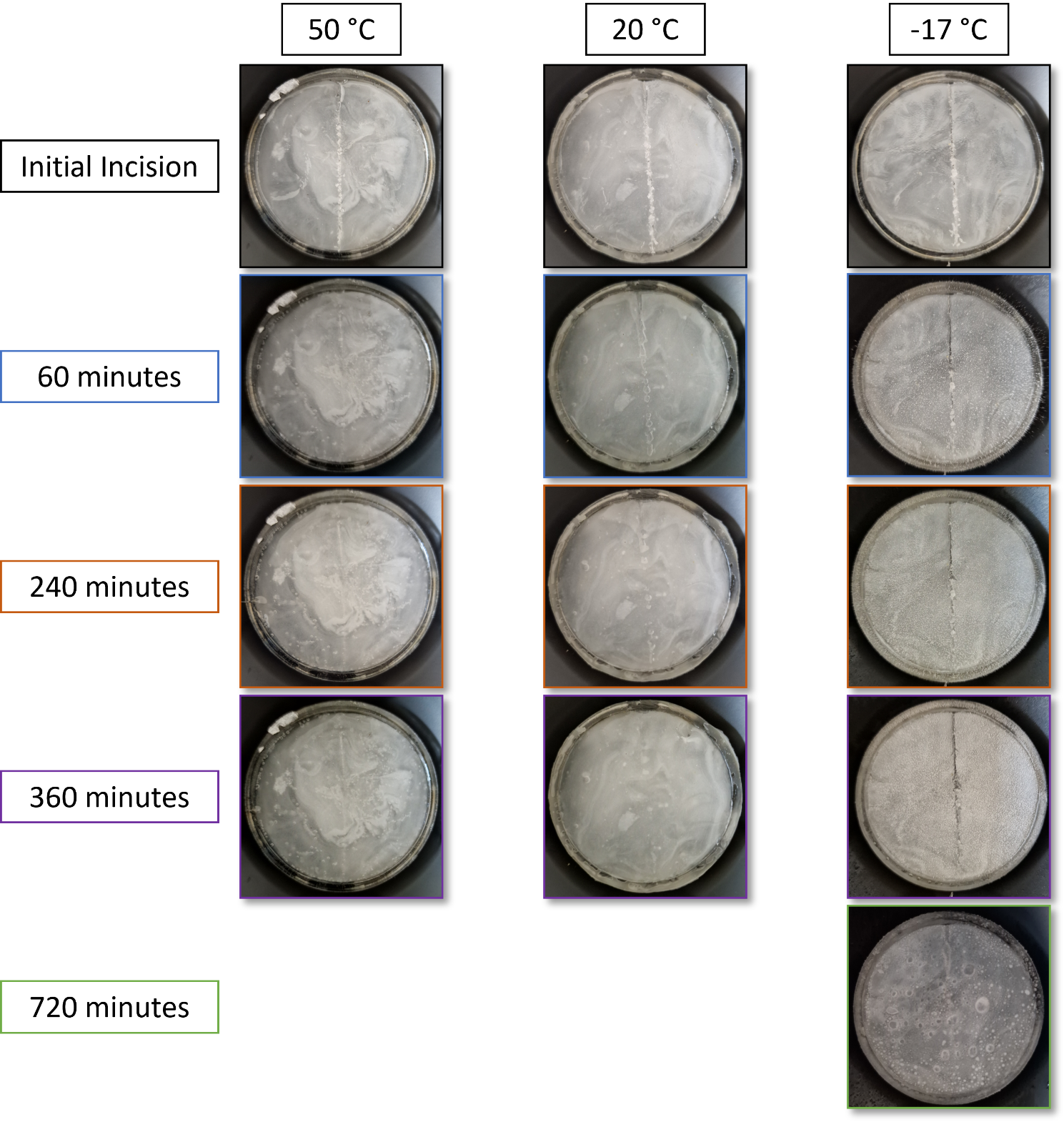  Supplementary Figure 7 Temperature dependence of self-healing mechanism of proposed PBDMS-based material. |
| --- |

To demonstrate the temperature dependence of the self-healing mechanism of the material, 10 g of PBDMS synthesised with kinematic viscosity of 50 cSt and a stoichiometric ratio $X_{\mathrm{BA}}^{\mathrm{st}}=0.8$, were positioned in a 25 mm diameter acrylic ring. Three environmental conditions were investigated to measure the efficiency of the material: high temperature (using a conventional oven regulated at 50 °C), room temperature (20 °C), and low temperature (-17 °C). An incision with the same dimensions was inflicted on the surface of each sample, and images of the samples were taken every two hours. As can be appreciated by the successive images in Supplementary Figure 6, the samples at room temperature and high temperature appeared to self-heal within six hours, while this was not held true for the sample kept below 0 °C. Upon a closer inspection, it is possible to observe that the high temperature accelerates the self-healing mechanism as the samples at 50 °C appeared fully healed within four hours, as suggested by the lack of a seam which is instead clearly still visible for the samples left to heal at room temperature (80% of healing). On the contrary, the sample left to heal at -17 °C, presented insignificant healing after four hours (below 10%), only partial healing by six hours (around 45%) and fully healed only after approximately twelve hours. The self-healing mechanism can be further appreciated in Supplementary Figure 8 below.

| 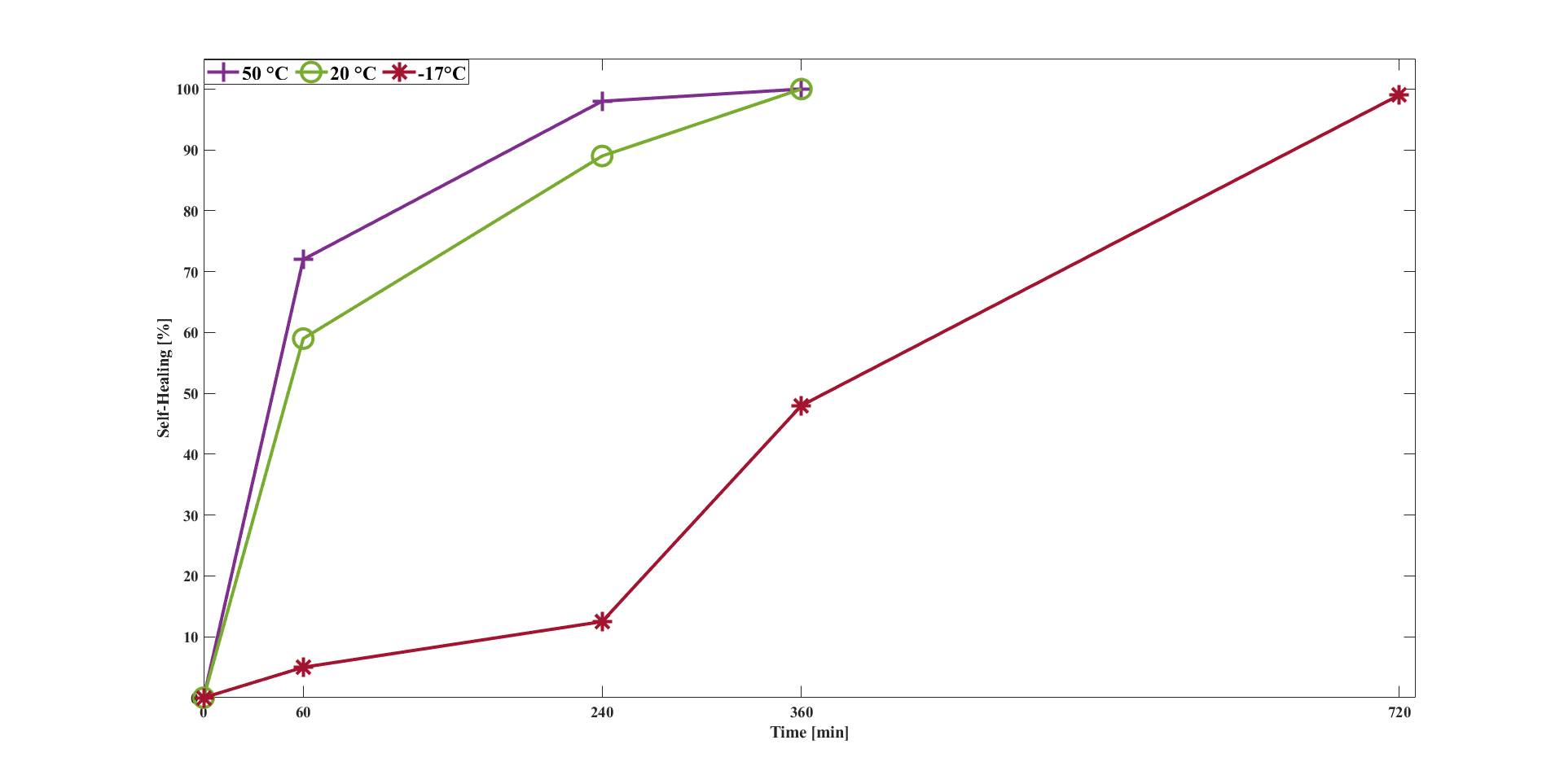  Supplementary Figure 8 Self-healing mechanism efficiency in respect to different temperature profiles. |
| --- |

In addition to the healing experiment when an incision was induced, oscillatory rheological measurements at different temperatures were conducted and included in the preliminary results in Supplementary Figure 9 below.

| 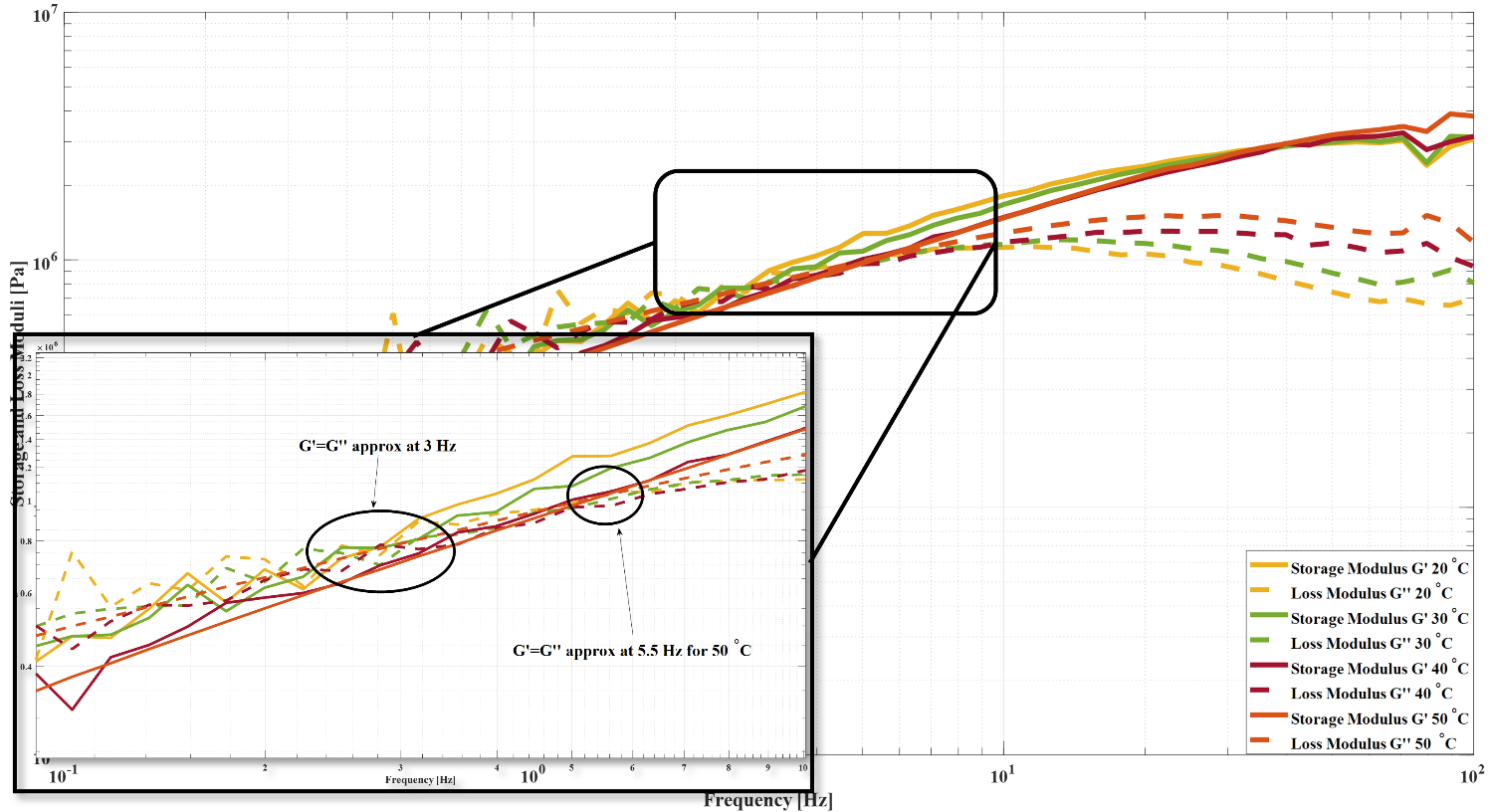  Supplementary Figure 9 Temperature controlled oscillatory rheological measurements of PBDMS between 25 and 50 °C. Continuous lines depict storage modulus (G') whereas dashed lines represent loss modulus (G''). Insert focuses on frequencies of interest. |
| --- |

In order to further investigate the dependence of the healing mechanism of the proposed material to temperature, oscillatory rheological measurements were performed with a 25mm parallel plate apparatus, with a temperature range spanning between 20 and 50 °C, at increments of 10 °C for each measurement. The PBDMS tested was synthesised from a PDMS precursor with kinematic viscosity of 50 cSt and a stoichiometric ratio $X_{\mathrm{BA}}^{\mathrm{st}}=0.8$. The range of investigated frequencies was from 0.1 to 100 Hz and the resulting storage (G’) and loss (G’’) moduli were drawn in logarithmic scales. As can be appreciated from the evolution of the curves of both moduli, raising the temperature profile of the measurements has a direct effect on both; the overall values of G’ appear to decline across the investigated frequencies, whereas those of G’’ increase with temperature, results that come in good agreement with literature [31-33]. The maximum values of G’ across all temperature profiles appeared at close proximity to each other. The insert in Figure 3 focuses on the point of intersection (G’=G’’) across all temperatures; for temperature profiles ranging from 20 to 40 °C this appears to be unaffected, however at 50 °C a clear shift towards higher frequencies is evident. This comes into good agreement with relative studies in literature [34, 35], as below the transition temperature of 43 °C, boric acid crosslinks behave as permanent links into the PDMS polymeric backbone. This shift of G’ = G’’ towards higher frequencies suggests decreased concentrations or lower molecular weight of the investigated polymer [36], demonstrating the effect of temperature to its properties, as above 43 °C the mobility of the polymeric network is dominated by that of the PDMS.

**References**

1. Li, X., et al., *Synthesis of polyborosiloxane and its reversible physical crosslinks.* Rsc Advances, 2014. **4**(62): p. 32894-32901.

2. Liu, Z., S.J. Picken, and N.A. Besseling, *Polyborosiloxanes (PBSs), synthetic kinetics, and characterization.* Macromolecules, 2014. **47**(14): p. 4531-4537.

3. Drozdov, F.V., et al., *Crosslinked polymers based on polyborosiloxanes: Synthesis and properties.* Journal of Organometallic Chemistry, 2019. **891**: p. 72-77.

4. Zhao, C., et al., *Anti-impact behavior of a novel soft body armor based on shear thickening gel (STG) impregnated Kevlar fabrics.* Smart Materials and Structures, 2019. **28**(7): p. 075036.

5. Wu, Q., et al., *Highly Stretchable and Self-Healing “Solid–Liquid” Elastomer with Strain-Rate Sensing Capability.* ACS Applied Materials & Interfaces, 2019. **11**(21): p. 19534-19540.

6. Zhao, C., et al., *Shear Stiffening Gels for Intelligent Anti-impact Applications.* Cell Reports Physical Science, 2020. **1**(12): p. 100266.

7. Cross, R., *Elastic and viscous properties of Silly Putty.* American Journal of Physics, 2012. **80**(10): p. 870-875.

8. Seetapan, N., et al., *Unimodal and bimodal networks of physically crosslinked polyborodimethylsiloxane: viscoelastic and equibiaxial extension behaviors.* Journal of Polymer Research, 2013. **20**(7): p. 1-9.

9. Jiang, W., et al., *Strain rate-induced phase transitions in an impact-hardening polymer composite.* Applied Physics Letters, 2014. **104**(12): p. 121915.

10. Zhang, S., et al., *Study the safeguarding performance of shear thickening gel by the mechanoluminescence method.* Composites Part B: Engineering, 2020. **180**: p. 107564.

11. Wang, Y., et al., *Dynamic behavior of magnetically responsive shear-stiffening gel under high strain rate.* Composites Science and Technology, 2016. **127**: p. 169-176.

12. Chang, Z., et al., *A Multifunctional Silly-Putty Nanocomposite Spontaneously Repairs Cathode Composite for Advanced Li−S Batteries.* Advanced Functional Materials, 2018. **28**(50): p. 1804777.

13. Zhang, D., et al., *Rheology of crosslinked entangled polymers: Shear stiffening in oscillatory shear.* Journal of Applied Polymer Science, 2020. **137**(9): p. 48421.

14. Tang, M., et al., *Synthesis of structure-controlled polyborosiloxanes and investigation on their viscoelastic response to molecular mass of polydimethylsiloxane triggered by both chemical and physical interactions.* Industrial & Engineering Chemistry Research, 2016. **55**(49): p. 12582-12589.

15. Christensen, R., *Theory of Viscoelasticity : an Introduction.* 2014.

16. Goertz, M., X.Y. Zhu, and J. Houston, *Temperature dependent relaxation of a “solid–liquid”.* Journal of Polymer Science Part B: Polymer Physics, 2009. **47**(13): p. 1285-1290.

17. Wang, S., et al., *Multifunctional polymer composite with excellent shear stiffening performance and magnetorheological effect.* Journal of Materials Chemistry C, 2014. **2**(34): p. 7133-7140.

18. Brummer, R., *Interpretation*, in *Rheology Essentials of Cosmetic and Food Emulsions*. 2006, Springer.

19. Seetapan, N., et al., *Unimodal and bimodal networks of physically crosslinked polyborodimethylsiloxane: viscoelastic and equibiaxial extension behaviors.* Journal of Polymer Research, 2013. **20**(7): p. 183.

20. Rogers, S., *Large amplitude oscillatory shear: Simple to describe, hard to interpret.* Physics Today, 2018. **71**(7): p. 34-40.

21. Liang, J. and X.-H. Zhang, *Rheological Properties of SP in Shock Transmission Application.* Journal of Materials in Civil Engineering, 2015. **27**(9): p. 04014250.

22. Barnes, H.A., *The yield stress—a review or ‘παντα ρει’—everything flows?* Journal of Non-Newtonian Fluid Mechanics, 1999. **81**(1): p. 133-178.

23. Allard, J. and N. Atalla, *Propagation of sound in porous media: modelling sound absorbing materials 2e*. 2009: John Wiley & Sons.

24. Chung, J. and D. Blaser, *Transfer function method of measuring in‐duct acoustic properties. I. Theory.* The Journal of the Acoustical Society of America, 1980. **68**(3): p. 907-913.

25. Boccaccio, M., et al., *Microperforated Panel and deep subwavelength Archimedean-inspired spiral cavities for multi-tonal and broadband sound absorption.* Applied Acoustics, 2021. **176**: p. 107901.

26. Maa, D.-Y., *Microperforated-panel wideband absorbers.* Noise control engineering journal, 1987. **29**(3): p. 77-84.

27. Testing, A.S.f. and Materials, *ASTM E 1050‐07. Standard Test Method for Impedance and Absorption of Acoustical Materials Using a Tube, Two Microphones and a Digital Frequency Analysis System*. 2007, Westcohocken, PA ASTM.

28. Barnard, A.R. and M.D. Rao, *Measurement of sound transmission loss using a modified four microphone impedance tube.* Proceedings of the ASME Noise Control and Acoustics Division (Noise-Con’04), 2004.

29. Yousefzadeh, B., et al., *An experimental study of sound transmission loss (STL) measurement techniques using an impedance tube.* Journal of the Acoustical Society of America, 2008. **123**(5): p. 3119.

30. Allan, P., et al., *Sound transmission testing of polymer compounds.* Polymer testing, 2012. **31**(2): p. 312-321.

31. Barnes, H.A., *The yield stress—a review or ‘παντα ρει’—everything flows?* Journal of Non-Newtonian Fluid Mechanics, 1999. **81**(1-2): p. 133-178.

32. Brummer, R., *Rheology essentials of cosmetic and food emulsions*. Springer.

33. Gutierrez-Lemini, D., *Engineering viscoelasticity*. 2014: Springer.

34. Goertz, M.P., X.-Y. Zhu, and J.E. Houston, *Temperature dependent relaxation of a “solid–liquid”.* Journal of Polymer Science Part B: Polymer Physics, 2009. **47**(13): p. 1285-1290.

35. Hailemariam, H. and G. Mulugeta, *Temperature-dependent rheology of bouncing putties used as rock analogs.* Tectonophysics, 1998. **294**(1): p. 131-141.

36. Sunthar, P., *Polymer Rheology*, in *Rheology of Complex Fluids*, J.M. Krishnan, A.P. Deshpande, and P.B.S. Kumar, Editors. 2010, Springer New York: New York, NY. p. 171-191.
